# Transitions in the flow patterns and aerodynamic characteristics of the flow around staggered rows of cylinders

**DOI:** 10.1371/journal.pone.0184169

**Published:** 2017-10-05

**Authors:** Shamsul Islam, Ghazala Nazeer, Zhou Chao Ying, Ziaul Islam, Raheela Manzoor

**Affiliations:** 1 COMSATS Institute of Information Technology Islamabad, Mathematics Department, Islamabad, Pakistan; 2 Shenzhen Graduate School, Harbin Institute of Technology Shenzhen University Town, Shenzhen, China; 3 Swedish Institute of Technology, Mechanical Engineering Department, Rawalpindi, Pakistan; 4 Mathematics Department, Sardar Bakhadur Khan Women’s University, Quetta, Baluchistan, Pakistan; Huazhong University of Science and Technology, CHINA

## Abstract

A two-dimensional numerical study of flow across rows of identical square cylinders arranged in staggered fashion is carried out. This study will unreveal complex flow physics depending upon the Reynolds number (R_e_) and gap spacing (g) between the cylinders. The combined effect of Reynolds number and gap spacing on the flow physics around staggered rows of cylinders are numerically studied for 20 ≤ R_e_ ≤ 140 and 1 ≤ g ≤ 6. We use the lattice Boltzmann method for numerical computations. It is found that with increase in gap spacing between the cylinders the critical Reynolds number for the onset of vortex shedding also increases. We observed a strong effect of Reynolds number at g = 2 and 4. Secondary cylinder interaction frequency disappears for large Reynolds number at g = 6 and 5 and the flow around cylinders are fully dominated by the primary vortex shedding frequency. This ensures that at large gap spacing with an increase in the Reynolds number the wakes interaction between and behind the cylinders is weaken. Furthermore, it also ensures that the wake interaction behind the cylinders is strongly influenced by the jets in the gap spacing between the cylinders. We also found that g = 2 is the critical gap spacing for flow across rows of staggered square cylinders for the considered range of Reynolds number. Depending on the Reynolds number we observed; synchronous, quasi-periodic-I, quasi-periodic-II, and chaotic flow patterns. In synchronous flow pattern, an in-phase and anti-phase characteristics of consecutive cylinders has been observed. The important physical parameters are also analyzed and discussed in detail.

## 1. Introduction

The complex flow physics in terms of wake structure mechanism and formation of two rows of staggered identical square cylinders has attracted researchers’ attention due to its importance in understanding industrial flows, for example, overhead power-line bundles, tubes in heat exchangers, chimney stacks, stays, bridge piers, flows around high-rise buildings, and offshore platforms, and many more. The vortex, gap flow switch, jets between gaps of cylinders, vortex shedding process, and combined frequencies are the important features of this flow, which is fascinating and complicated. These phenomena are strongly dependent upon the Reynolds numbers (R_e_ = U_∞_d/υ, where U_∞_ is the uniform inflow velocity, d is the size of the cylinder and υ is the kinematic viscosity of the fluid) and gap spacing (g = s/d, where s is the surface-to-surface distance between the cylinders). For example, due to aerodynamic interference [1], the collapse of three (out of eight) natural draft cooling towers at Ferry bridge (UK) in 1965, has attracted researchers interest around such kind of problems. In addition, it is important to understand the dramatic change in forces and downstream evolution of vortex frequencies on each cylinder.

In spite the fact that the flow around square cylinders has great relevance to practical engineering applications, it has received much less attention as compared to the flow around circular cylinders. It is true that such real-life cases are often characterized by large three-dimensional turbulent fluctuations. But important information regarding basic flow behavior around two rows of staggered square cylinders can still be gleaned at a low Reynolds numbers for clear understanding. Computations of square cylinder can reduce number of uncertainties associated with experimental investigations of a cylinder wake, such as vibration, finite radius of curvature at the edges, turbulence intensity of the incoming flow and the surface roughness of the cylinder. The square geometry has some computational advantages, and due to its round shape it can be easily represented in Cartesian coordinates. To the best of our knowledge, numerical studies of the effect of Reynolds numbers on flow across two rows of staggered square cylinders have not been reported in literature.

There have been a number of investigations on the wake of two, three, five and row of side-by-side circular [2–9] and square [10–20] cylinders. Bearman and Wadcock [2] experimentally studied the flow past a pair of circular cylinders. Chauve and Le Gal [3] studied in detail the wakes behind a row of sixteen cylinders at (R_e_, g) = (80, 3). They observed that some of the wakes intermittently stop oscillation behind the cylinders. Guillaume and LaRue [4] examined relatively narrow and wide wakes behind three to five cylinders with g = 0.75 and R_e_ = 2500. Gu and Sun [5] experimentally studied the interference effect of two identical circular cylinders in staggered arrangement for high Reynolds numbers. Zhang and Zhou [6] experimentally examined the effect of unequal gap spacing around three side-by-side circular cylinders for Reynolds number ranging from 150 to 2000. Kang [7] numerically studied the effect of gap spacing for flow past three circular cylinders and observed different flow regimes at R_e_ = 100. Zhou *et al*. [8] examined experimentally the effect of Reynolds number (R_e_ = 1.5 × 10^3^ to 2 × 10^4^) of flow past two staggered circular cylinders. They discussed in detail the variation of Strouhal numbers. Yan *et al*. [9] experimentally and numerically studied the characteristics of flow around three staggered circular cylinders using the multiple-relaxation-time lattice Boltzmann method (MRT-LBM). They found the steady and unsteady flow regions behind the upstream cylinder by varying the gap spacing between the cylinders from 1 to 10 at a fixed Reynolds number (R_e_ = 200).

Mizushima and Takemoto [10] numerically examined that each jet between the square cylinders are independent of each other for large gap spacing between the cylinders. However, at smaller gap spacing between the cylinders confluence of several jets occurs. Agrawal *et al*. [11] numerically studied the flow around two side-by-side square cylinders and observed the synchronous and chaotic flow patterns at R_e_ = 73. Sewatkar *et al*. [12] carried out numerical simulation of the flow past a row of square cylinders. They studied the combined effects of Reynolds number and gap spacing between the cylinders for 30 ≤ R_e_ ≤ 140 and 1 ≤ g ≤ 4. They found that the critical Reynolds number for the onset of vortex shedding increases with increase in gap spacing between the cylinders. Furthermore, they found the synchronous, quasi-periodic-I and quasi-periodic-II flow patterns. Moussaoui *et al*. [13] numerically examined the flow characteristics and heat transfer past three staggered square cylinders using the MRT-LBM. They studied the combined effect of Reynolds numbers (10 ≤ R_e_ ≤ 100) and gap spacing (1 ≤ g ≤ 2) between the cylinders. They did not study the jet form between the cylinders and the presence of primary and secondary cylinder interaction frequencies for smaller and larger gap spacings. Alam and Zhou [14] experimentally examined the wake structure mechanism behind two identical side-by-side square cylinders at R_e_ = 300 and 1 ≤ g ≤ 5. They discussed in detail the gap vortices, flow switch, merging of two streets into one and stability. Furthermore, they found single bluff body, narrow and wide streets, transition and the coupled-sheet flow patterns. Abbasi *et al*. [15] examined the effect of Reynolds number on flow past four square cylinders in an inline square configuration and discussed in detail the observed flow patterns. Chatterjee and Biswas [16] reported the chaotic and synchronous flow patterns at 1 ≤ g ≤ 5 for a fixed Reynolds number of 100. They argued that these flow patterns are the results of primary vortex shedding frequency and secondary cylinder interaction frequency for flow around two rows of staggered cylinders using Finite volume based computational fluid dynamics (CFD) solver using PISO algorithm. One can find the effect of Reynolds numbers and gap spacing for flow past three side-by-side square cylinders numerically [17–19]. These researchers discussed the effect of Reynolds numbers and gap spacing, and observed various flow patterns. Kumar *et al*. [20] numerically studied quasi-periodic, synchronous, and chaotic flow patterns at 0.3 ≤ g ≤ 12 for a fixed Reynolds number equals to 80.

The above literature shows that not much attention has been paid to flow past two rows of staggered square cylinders; in particular, the effect of Reynolds numbers has not been studied. This study aims to partially fill this gap in literature. Chatterjee and Biswas [16] have investigated the different flow patterns and there study is limited to a single Reynolds number and fixed the stream-wise gap spacing between the cylinders. The main goal of the present study is to systematically investigate the different flow patterns by changing the Reynolds number. We show the effect of Reynolds number from 20 to 140 with 1 ≤ g ≤ 6. The critical Reynolds number for the onset of vortex shedding frequency for the proposed problem is also discussed for the first time. In literature merging of jets [11, 12, 20] and variation in the wake size [13, 14, 17, 18, 19] has been considered separately whereas we discuss in detail the effect of jets between the cylinders on the wake interaction.

In Section 2 we provide problem description and numerical details. The grid independence, effect of the computational domain, and a validation are provided in Section 3 based on the available data of the single and two side-by-side square cylinders in literature. The dependence of flow patterns on R_e_ and g is presented and discussed in Section 4 systematically. The main findings of this work are finally concluded in Section 5.

## 2. Problem description and numerical details

**Fig 1:**
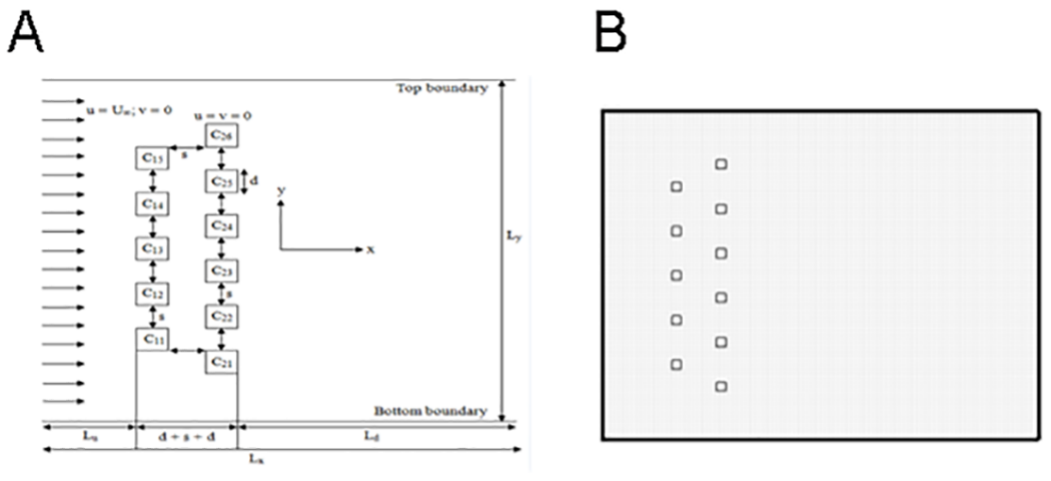
(A) Schematic configuration of the flow around two staggered rows of square cylinders. (B) The computational mesh around the cylinders for the case of (R_e_, g) = (140, 4).

The purpose of this study is to simulate and analyze the flow characteristics of flow past two staggered rows of square cylinders Fig 1(A). The cylinders are fixed and identical in size (d) and shape. The spacing (s) between the cylinders is varied (g = s/d = 1, 2, 3, 4, 5 and 6) and the Reynolds numbers from 20 to 140. The upstream (C_11_, C_12_, C_13_, C_14_ and C_15_) and downstream (C_21_, C_22_, C_23_, C_24_, C_25_ and C_26_) rows have comprised five and six cylinders, respectively. The cylinders have been marked as C_21_ through C_26_ starting from the bottom of the computational domain. The stream-wise and the transverse directions with the origin of the coordinate system are x and y, respectively. The length of the computational domain L_x_ = L_u_ + d + s + d + L_d_ (where L_u_ = 8d is the upstream distance from the channel inlet and L_d_ = 35d is the downstream distance from the downstream row of cylinders rear to exit of the domain) is taken as 45d. The height (L_y_) of the computational domain is 11d. The velocity vectors are represented by **u** = (u, v). The simulations for the gap spacing 1, 2, 3, 4, 5 and 6 have been carried out using grids of sizes 441 × 921, 541 × 941, 641 × 961, 741 × 981, 861 × 1001, and 981 × 1021, respectively. An overall mesh at (R_e_, g) = (140, 4) is shown in Fig 1(B) as a representative case.

A uniform inflow velocity (u = U_*∞*_; v = 0) is prescribed at the inlet boundary of the channel. The convective boundary condition (∂_*t*_u + *U*_∞_∂_*x*_u = 0) is applied at the exit of the computational domain [21]. The exit of the computational domain is far enough from the cylinders to ensure that there are no effects of the unconstrained movement of fluid. The periodic boundary conditions [22] have been applied on the bottom and top boundaries of the computational domain. No-slip boundary conditions (u = v = 0) are applied on the surfaces of the cylinders [21]. The forces on the surfaces of the cylinders are calculated using the momentum exchange method [23]. The simulation starts with the condition that the flow is initially at rest. The initial transient variations of physical parameters such as mean drag coefficient (C_dmean_) and Strouhal number (S_t_ = f_s_d/U_∞_, where f_s_ is the vortex shedding frequency) are not included in the obtained results. The S_t_ is computed by performing Fast Fourier Transform (FFT) on the lift coefficient.

Two-dimensional lattice Boltzmann method (LBM) is used in this particular study. For an incompressible unsteady flow, the flow field can be described by the continuity equation and momentum equations,

∇.ρu=0, (1)

$$\rho \left(\frac{\partial u}{\partial t}+(u.\nabla )u\right)=-\nabla p+\rho \upsilon \nabla^{2}u.$$ (2)

where **u**, ρ, p and t are the flow velocities, density, pressure and time, respectively. Applying the Chapman-Enskog expansion [24] with the restriction of low Mach number the above Eqs (1) and (2) can be obtained by the discrete lattice Boltzmann Eq (3)

$${\text{f}}_{\text{i}}(\text{x}+{\text{e}}_{\text{i}}\Delta \text{t},\text{t}+\Delta \text{t})={\text{f}}_{\text{i}}(\text{x},\text{t})-({\text{f}}_{\text{i}}-{\text{f}}_{\text{i}}^{\left(\text{eq}\right)})/\tau .$$ (3)

where f_i_(**x**, t) is the distribution function which indicates the position **x** of a particle at time t, Δt is the time step, ${\text{f}}_{\text{i}}^{\left(\text{eq}\right)}$ is the equilibrium distribution function and τ is the single relaxation time parameter which control the stability of the method. The equilibrium distribution function is defined by:

$${\text{f}}_{\text{i}}^{\left(\text{eq}\right)}=\rho \omega_{\text{i}}[1+3({\text{e}}_{\text{i}}.\text{u})+4.5{\left({\text{e}}_{\text{i}}.\text{u}\right)}^{2}-1.5{\text{u}}^{2}].$$ (4)

where the weighting coefficient ω_i_ are

$$\omega_{\text{i}}=\{\begin{array}{cc} 4/9, & \text{i}=0\\ 1/9, & \text{i}=1,2,3,4\\ 1/36, & \text{i}=5,6,7,8 \end{array}$$

The kinematic viscosity ʋ can be obtained in the following way:

$$\upsilon ={\text{c}}_{s}^{2}(\tau -0.5)\;{\left(\Delta \text{x}\right)}^{2}/\Delta \text{t}.$$ (5)

The pressure, density and flow velocity can be obtained by

$$\text{p}=\rho c_{s}^{2},$$ (6)

$$\rho =\sum_{i=0}^{8}{\text{f}}_{\text{i}},$$ (7)

$$\text{u}=\frac{1}{\rho }\sum_{i=1}^{8}{\text{e}}_{\text{i}}{\text{f}}_{\text{i}}.$$ (8)

The chosen range of Reynolds number fulfills the two-dimensionality requirements. In LBM, the movement of a large number of particles on a lattice is the basic proposition. In this study we have used D2Q9 (where D and Q denotes the dimensions and number of velocity particles, respectively) LBM model. Basically, LBM consist of two main steps: (i) streaming (left hand side of Eq (3)) and (ii) collision (right hand side of Eq (3)). The collisions take place between particles at each time step and their velocities change their directions but, the net mass and momentum are conserved. The boundary conditions are applied after the streaming step and the entire process is solved iteratively until the convergence is ensured by the following relation

$$\frac{\sqrt{\sum_{\text{l},\text{m}}{\left[{\text{u}}_{\text{l},\text{m}}^{\left(\text{k}+1\right)}-{\text{u}}_{\text{l},\text{m}}^{\left(\text{k}\right)}\right]}^{2}}}{\sqrt{\sum_{\text{l},\text{m}}{\left[{\text{u}}_{\text{l},\text{m}}^{\left(\text{k}+1\right)}\right]}^{2}}}\leq 1\times 10^{-6}.$$ (9)

The LBM is found to be a second-order accurate method in space and conditionally stable depending on the single-relaxation-time parameter ‘τ’. The most important advantage of this method is the ease of introducing the obstacle in the flow which makes this method fully suited for the present study. Here, the equilibrium distribution is found to be valid only for small Mach numbers. Also the density function is represented by particles that move one lattice length at every time step. Thus the speed of sound calculated from the diffusion velocity of particles therefore is always larger than the macroscopic velocity which results in small Mach number restriction. The lattice Boltzmann method actually belongs to a class of the pseudo-compressible solvers of the Navier-Stokes equations for incompressible fluid flow. In order to correctly simulate the incompressible fluid flow, it must be ensure that the Mach number, M_a_ = U_∞_/C_s_<< 1. Furthermore, low Mach number requires that the uniform inflow velocity U_∞_ be sufficiently small.

The present computational code is originally written and edited using the Intel Fortran platform with 64-bits. All the computations were performed on an Intel i7-2600, four core 3.2GHz, 4GB DDR3 memory, 320GB hard disk computer, with a Window 7 (64-bits) system. The present simulation adopts the uniform and square grid. More details can be seen in review article of Chen and Doolan [25]; books of Sukop and Thorne [22]; Succi [26] and Kruger *et al*. [27], and research articles [28, 29].

## 3. Grid independence, effect of computational domain and code validation study

**Fig 2:**
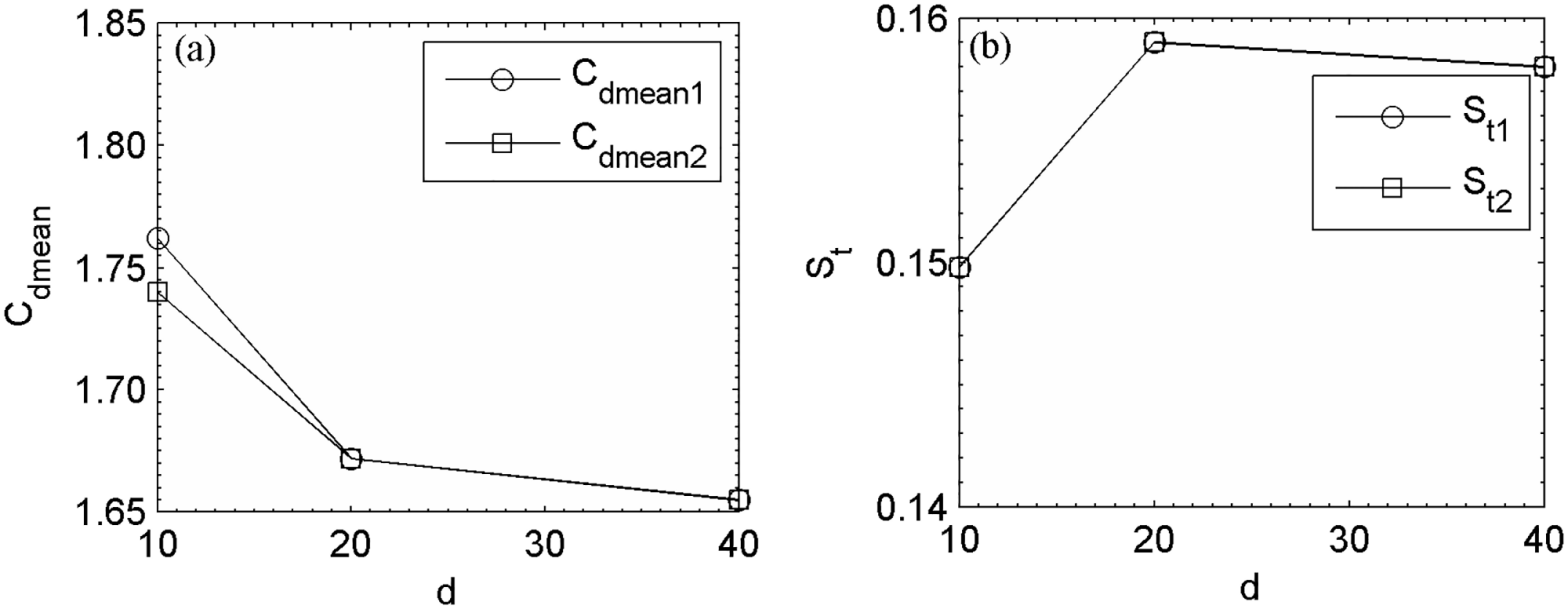
The effect of grid points on the flow past two side-by-side square cylinders at (R_e_, g) = (73, 4) in terms of (a) mean drag coefficients and (b) Strouhal numbers.

The basic validation of this present computational code, basics of lattice Boltzmann method, grid independence study and effect of computational domain has already been carried out for flow past a rectangular cylinder [30], and flow past more than two side-by-side cylinders [18, 19, 28, 29]. In Fig 2(a) and 2(b) and Tables 1 and 2 the C_dmean1_ and C_dmean2_ are the mean drag coefficients of the lower and upper cylinders, respectively, in two side-by-side square cylinders arranged from bottom to top. Similarly, S_t1_ represents the Strouhal number of lower, and S_t2_ represents the Strouhal number of upper cylinders. The drag and lift coefficients, C_d_ and C_l_, are obtained by normalizing the forces by 2F_d_/ρU^2^_∞_ and 2F_l_/ρU^2^_∞_ (ρ is the fluid density, F_d_ is the force along stream-wise direction and F_l_ is the force along cross-stream-wise direction).

**Table 1:** The effect of computational domains on the flow past two side-by-side square cylinders at (R_e_, g) = (73, 4).

Cases	L_x_ × L_y_	C_dmean1_	C_dmean2_	S_t1_	S_t2_
I	L_u_ = 6d; L_d_ = 35d; L_y_ = 11d	1.6952 (1.2%)	1.6952 (1.2%)	0.1552 (2.5%)	0.1552 (2.5%)
II	L_u_ = 8d; L_d_ = 35d; L_y_ = 11d	1.6752	1.6752	0.1591	0.1591
III	L_u_ = 12d; L_d_ = 35d; L_y_ = 11d	1.6714 (0.3%)	1.6714 (0.3%)	0.1591 (0.0%)	0.1591 (0.0%)
IV	L_u_ = 8d; L_d_ = 25d; L_y_ = 11d	1.6848 (0.6%)	1.6848 (0.6%)	0.1594 (0.2%)	0.1594 (0.2%)
V	L_u_ = 8d L_d_ = 45d; L_y_ = 11d	1.6698 (0.3%)	1.6698 (0.3%)	0.1591 (0.0%)	0.1591 (0.0%)
VI	L_u_ = 8d; L_d_ = 35d; L_y_ = 8d	1.6932 (1.1%)	1.6932 (1.1%)	0.1566 (1.6%)	0.1566 (1.6%)
VII	L_u_ = 8d; L_d_ = 35d; L_y_ = 14d	1.6702 (0.3%)	1.6702 (0.3%)	0.1594 (0.2%)	0.1594 (0.2%)

**Table 2:** The aerodynamic force characteristics of flow past a single square cylinder at different Reynolds numbers.

R_e_		C_dmean_	S_t_
75	Present	1.4086	0.1392
	Sohankar *et al*. [32]	1.5090	0.1330
	Gera *et al*. [33]	1.5240	0.1220
100	Present	1.4236	0.1498
	Sohankar *et al*. [32]	1.4440	0.1450
	Gera *et al*. [33]	1.4610	0.1290
120	Present	1.4150	0.1542
140	Present	1.4020	0.1565

A grid independence study was performed by comparing the calculated flow characteristics using 10, 20 and 40 points to discretize the cylinder side for flow past two side-by-side square cylinders. A maximum difference of 1.3%, 1.3%, 0.6% and 0.6% on C_dmean1_, C_dmean2_, S_t1_ and S_t2_, respectively, was found between the last two cases. In this study, we adopt a resolution of 20 points for square cylinder. In addition, we choose 20 points, keeping in mind the constraints of long running simulations and computational resources. Kumar *et al*. [20] found that 16–20 points cylinder resolution is enough to obtain grid independent results. We also found that L_u_ = 8d, L_d_ = 35d and L_y_ = 11d is the best choice in terms of computational length see Table 1.

**Fig 3:**
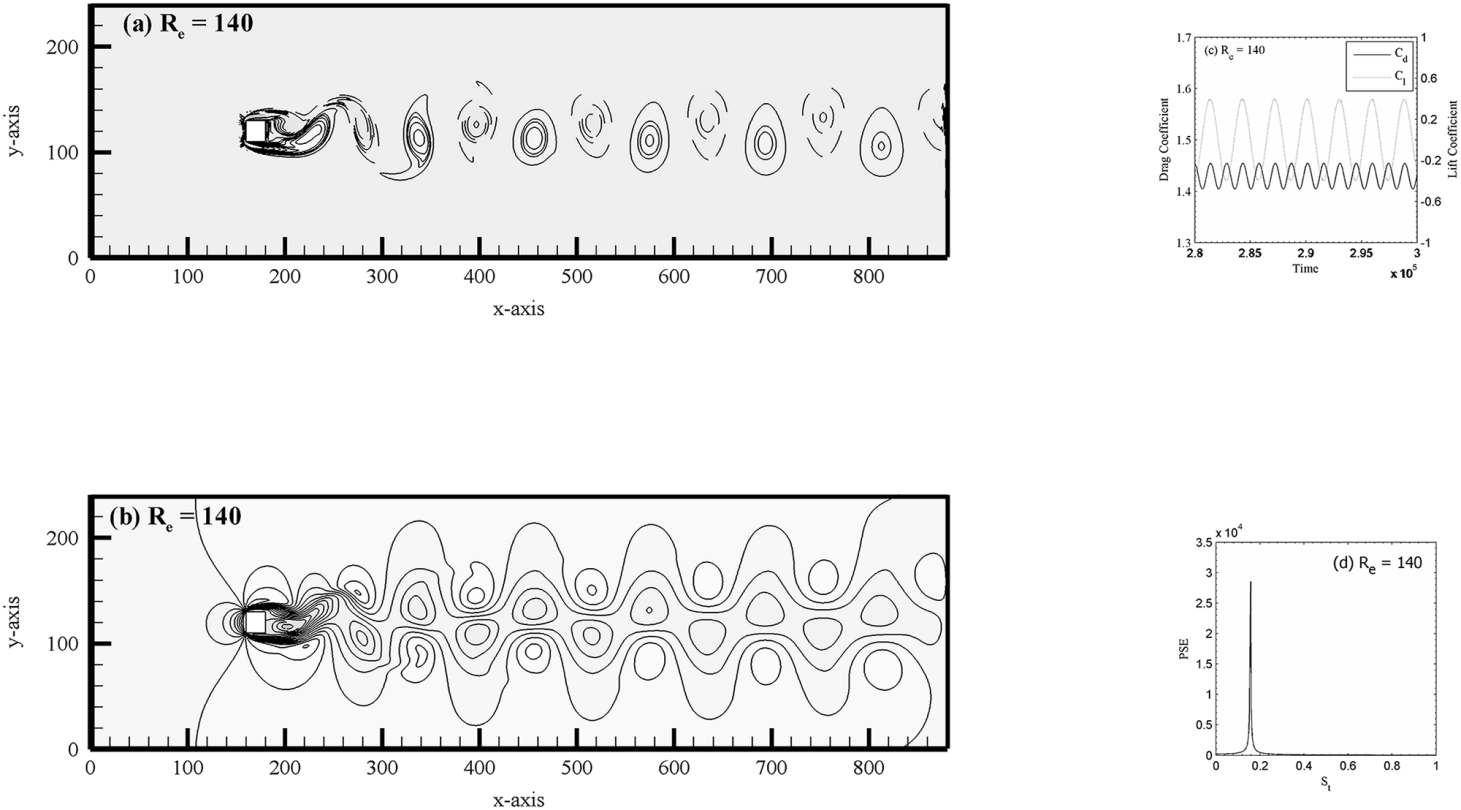
(a) Vorticity contours visualization, (b) velocity profile, (c) drag and lift coefficients and (d) power spectra analysis of flow past a single square cylinder.

In addition, for the sake of the present numerical study, we have carried out a separate validation study for flow past a single square cylinder see Fig 3(a), 3(b), 3(c) and 3(d) and Table 2 and two side-by-side square cylinders (see Table 3) at (R_e_, g) = (73, 4). It is to be noted that in case of single square cylinder in a channel we adopted the no-slip [21] boundary conditions on the top and bottom walls of the channel. The same boundary conditions and channel length adopted as discussed already for the proposed problem in Section 2. The vortex pattern in the wake behind the cylinder shown in Fig 3(a) composes of negative and positive alternate shed vortices, i.e. the well-known Karman vortex street at R_e_ = 140. The velocity profile is also presented in Fig 3(b). It is observed that the drag and lift coefficients are settles to a periodic behavior Fig 3(c). Furthermore, no other peaks observed in the power spectra analysis of lift coefficient for R_e_ = 140 in Fig 3(d). In this paper in spectra plots ‘PSE’ stands for power spectrum energy. These findings are in good agreement with those observed by Davis and Moore [31]. Similar characteristics observed for R_e_ = 75, 100 and 120 (not shown). The force statistics data of different Reynolds numbers are given in Table 2 with available data in the literature [32, 33]. These findings further gives us the confidence that the present code recovered correctly all the silent features of an isolated cylinder in a cross-flow. The comparison of present results and those of Agrawal *et al*. [11] are given in Table 3. The minor discrepancy in S_t_ found could be due to the difference in the cylinder size and computational domain, etc. used.

**Table 3:** Comparison of present numerical results and Agrawal *et al*. [11] for flow past two side-by-side square cylinders at R_e_ = 73.

g		C_dmean1_	C_dmean2_	S_t1_	S_t2_
1	Present	1.8668	1.8865	0.1520	0.1520
	Agrawal *et al*. [11]	1.9000	1.9000	0.1750	0.1750
1.5	Present	1.8525	1.8281	0.1680	0.1680
	Agrawal *et al*. [11]	1.9000	1.9000	0.1620	0.1620
2	Present	1.7880	1.7822	0.1622	0.1622
	Agrawal *et al*. [11]	1.8000	1.8000	0.1650	0.1650
4	Present	1.6752	1.6752	0.1591	0.1591
	Agrawal *et al*. [11]	1.7500	1.7500	0.1660	0.1660
6	Present	1.6215	1.6215	0.1582	0.1582
	Agrawal *et al*. [11]	1.7000	1.7000	0.1650	0.1650

## 4. Results and discussions

To the authors’ knowledge, there are no experimental measurements and numerical studies of the flow patterns for the problem under consideration in the open literature to compare with the numerical work of this study. We will mostly compare our results with flow past a single, two, three, and row of square cylinders available in the literature. Results are presented for two-dimensional computations for 75 ≤ R_e_ ≤ 140 and 1 ≤ g ≤ 6 using the single-relaxation-time lattice Boltzmann method. In this study C_12_, C_13_, C_23_, and C_24_ of two consecutive cylinders (arbitrarily chosen) for time history analysis of drag and lift coefficients, and power spectra of lift coefficients to further verified the shedding pattern of observed flow patterns. In vorticity contours visualization plots the solid and dashed lines represent the positive and negative shed vortices generated from the lower and upper corners of the cylinders. We used the solid and dotted lines in drag and lift coefficients plots. The solid line used in power spectra graphs for chosen cylinders in this study.

**Fig 4:**
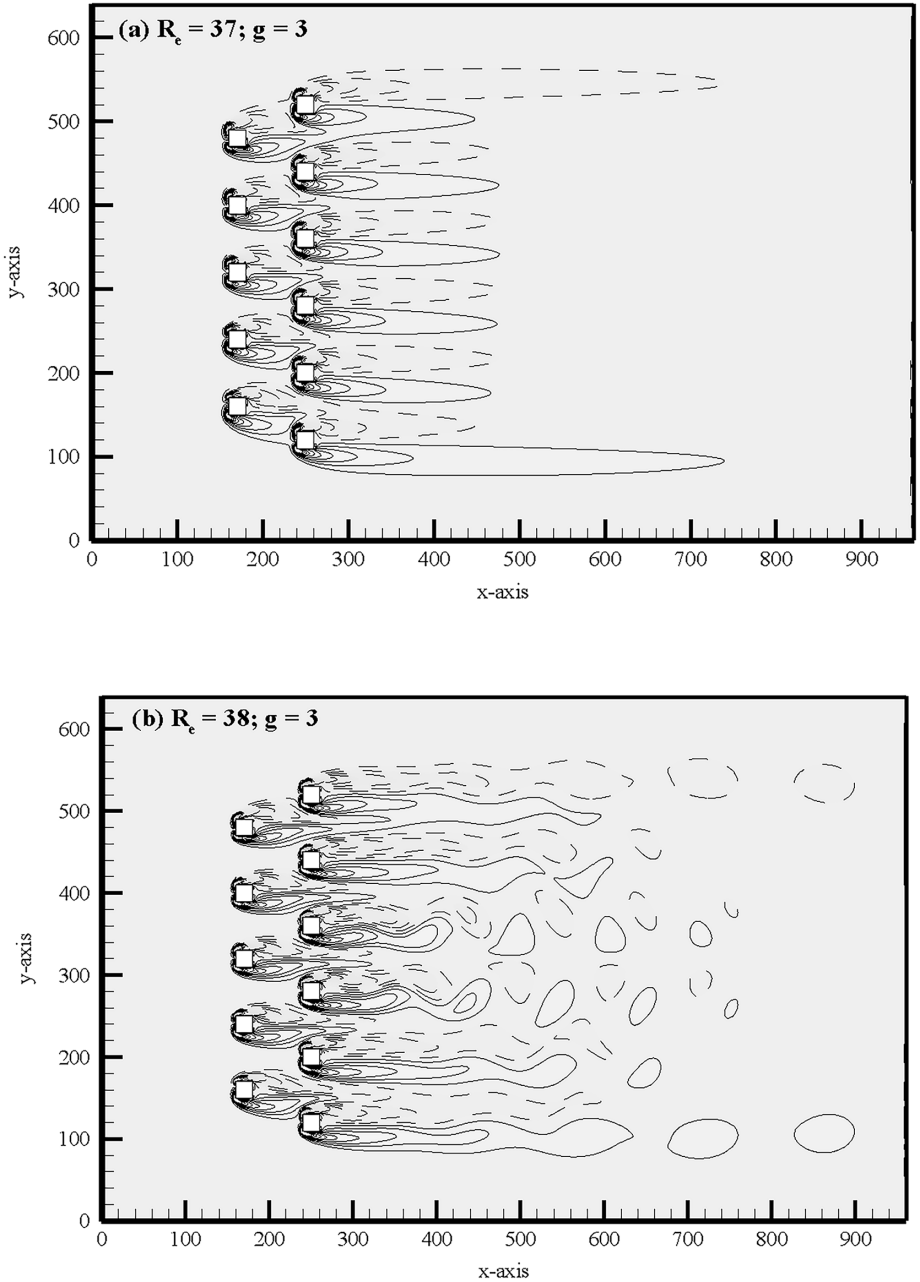
Onset of vortex shedding of flow from steady to unsteady at (a) R_e_ = 37 and g = 3 and (b) R_e_ = 38 and g = 3.

**Fig 5:**
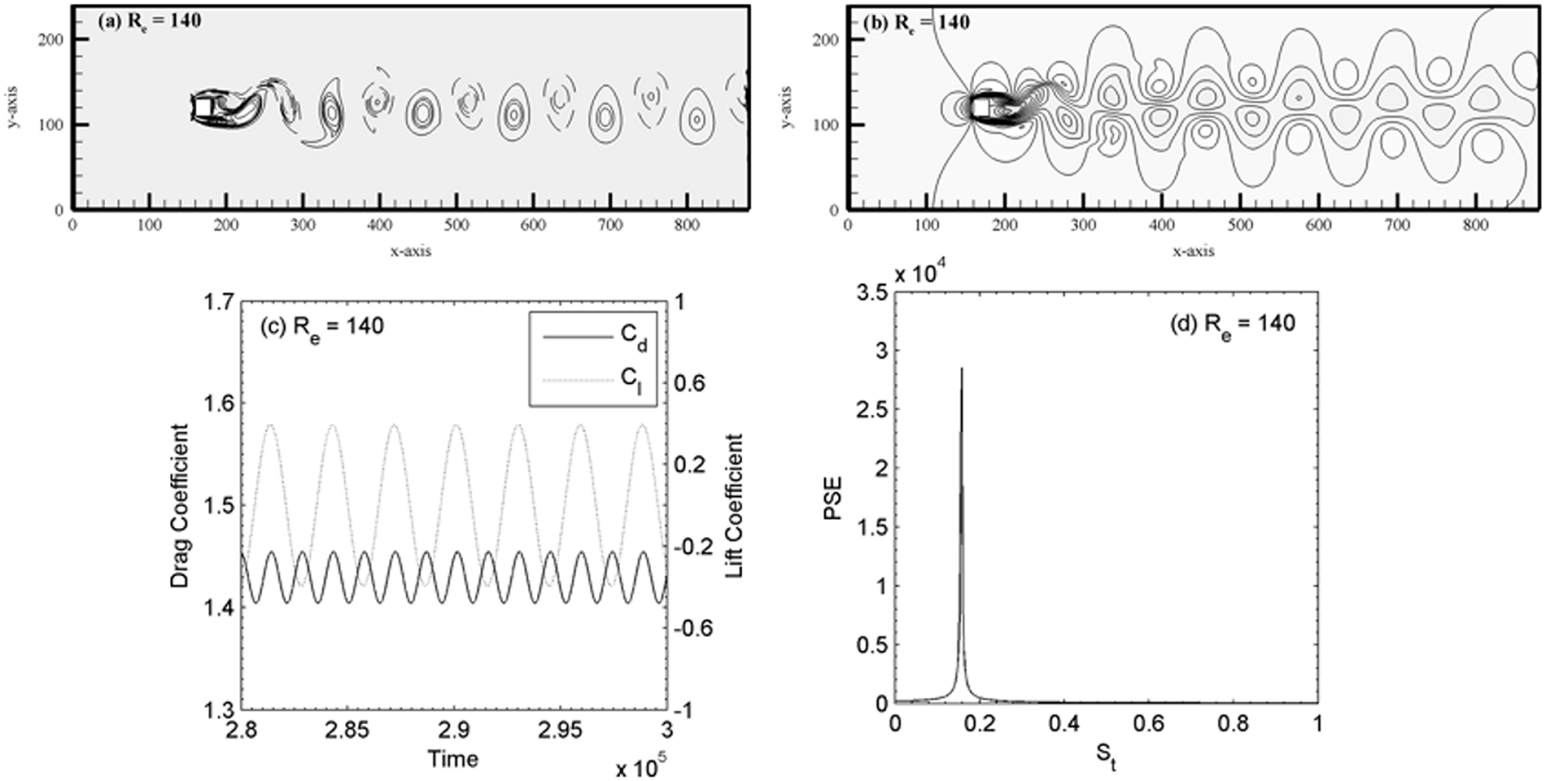
Variation of critical Reynolds number for flow past the two rows of staggered square cylinders with gap spacing.

One typical example of onset of vortex shedding of flow from steady to unsteady is shown in Fig 4(a) and 4(b) at g = 3 for two different Reynolds numbers. Our study found that the maximum value of critical Reynolds number (R_ecr_) is 41.5 at g = 6, and reduces with a reduction in gap spacing see Fig 5. It is to be noted that to find the critical Reynolds number for each gap spacing we start from R_e_ = 20 and increase it with the increment of 0.5 until we not find the critical value. This is actually due to increase in the vorticity production from the boundary layers with decreasing the separation between the cylinders as a result of gap induced accelerated effects. With a reduction in gap spacing the vorticity generation increased is the main cause of the reduction of critical Reynolds numbers with increasing the gap spacing [12]. Therefore, the lower limit of Reynolds number for further analysis is set at 75. Saha *et al*. [34] observed that the flow loses its two-dimensionality between 150 ≤ R_e_ ≤ 175, therefore the upper limit of R_e_ in this study has been decided as 140.

**Fig 6:**
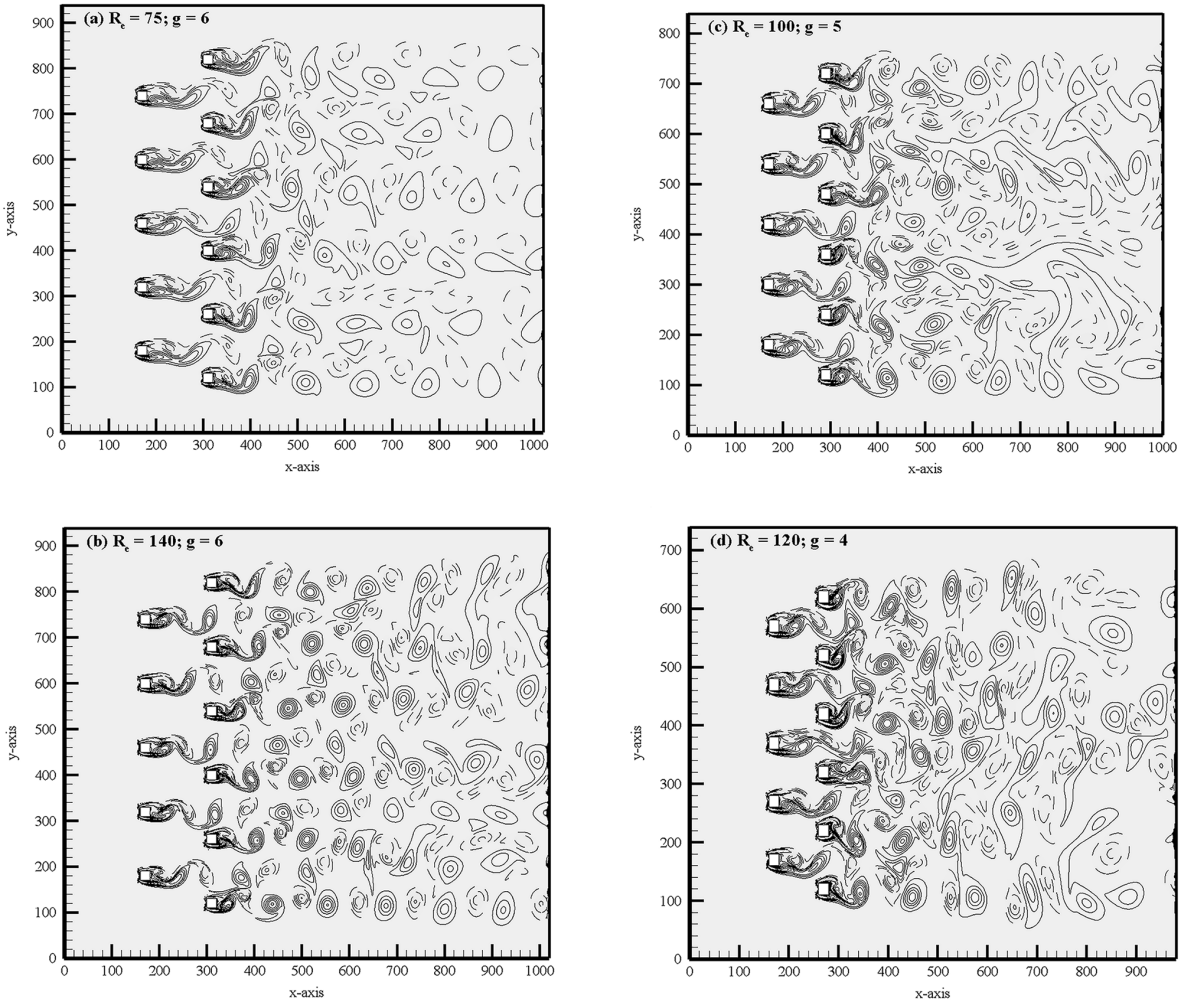
Instantaneous vorticity contours visualization corresponding to synchronous flow pattern for (a) (R_e_, g) = (75, 6), (b) (R_e_, g) = (140, 6), (c) (R_e_, g) = (100, 5) and (d) (R_e_, g) = (120, 4).

**Fig 7:**
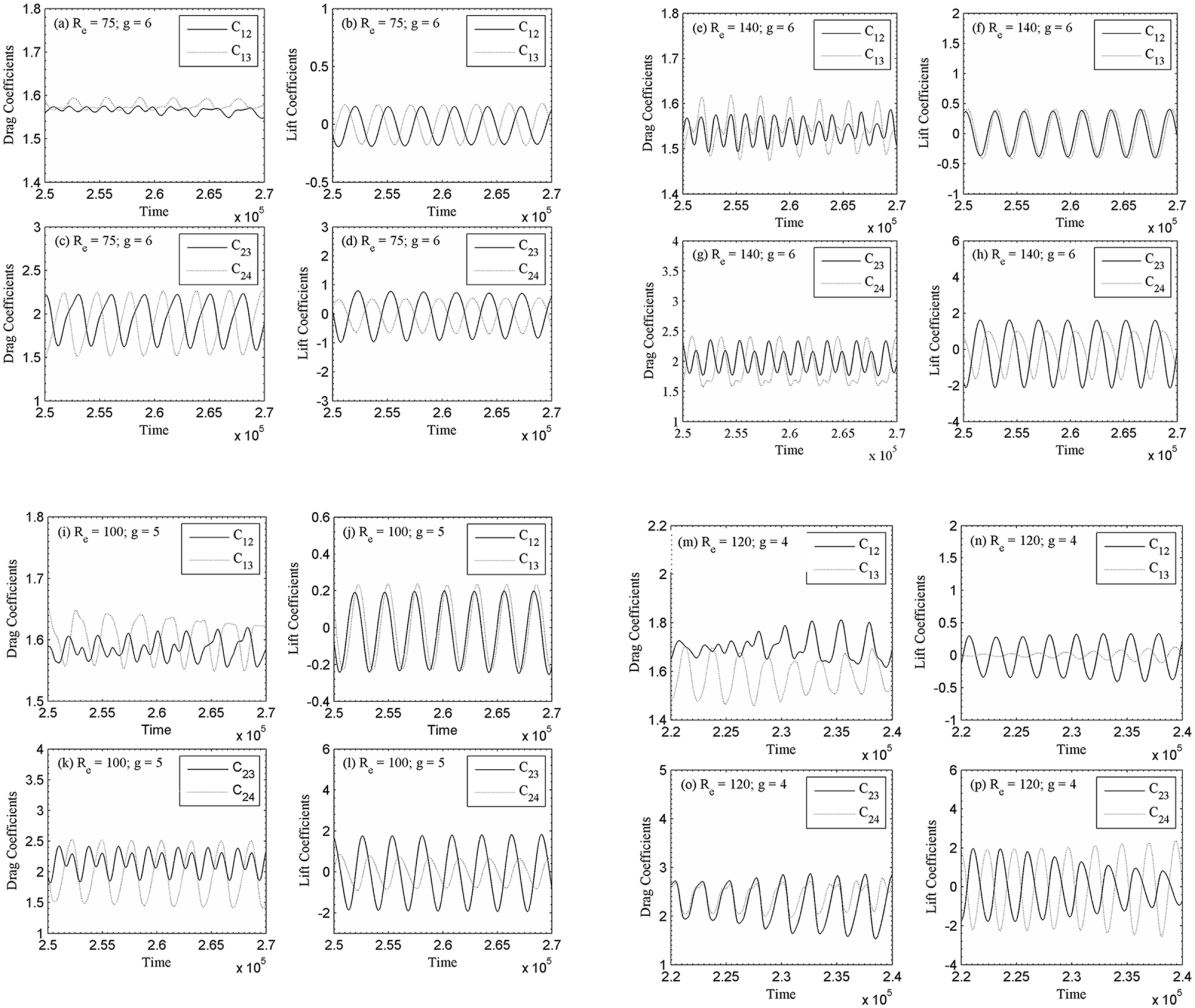
Time-history analysis of drag and lift coefficients (a-d) (R_e_, g) = (75, 6), (e-h) (R_e_, g) = (140, 6), (i-l) (R_e_, g) = (100, 5) and (m-p) (R_e_, g) = (120, 4).

**Fig 8:**
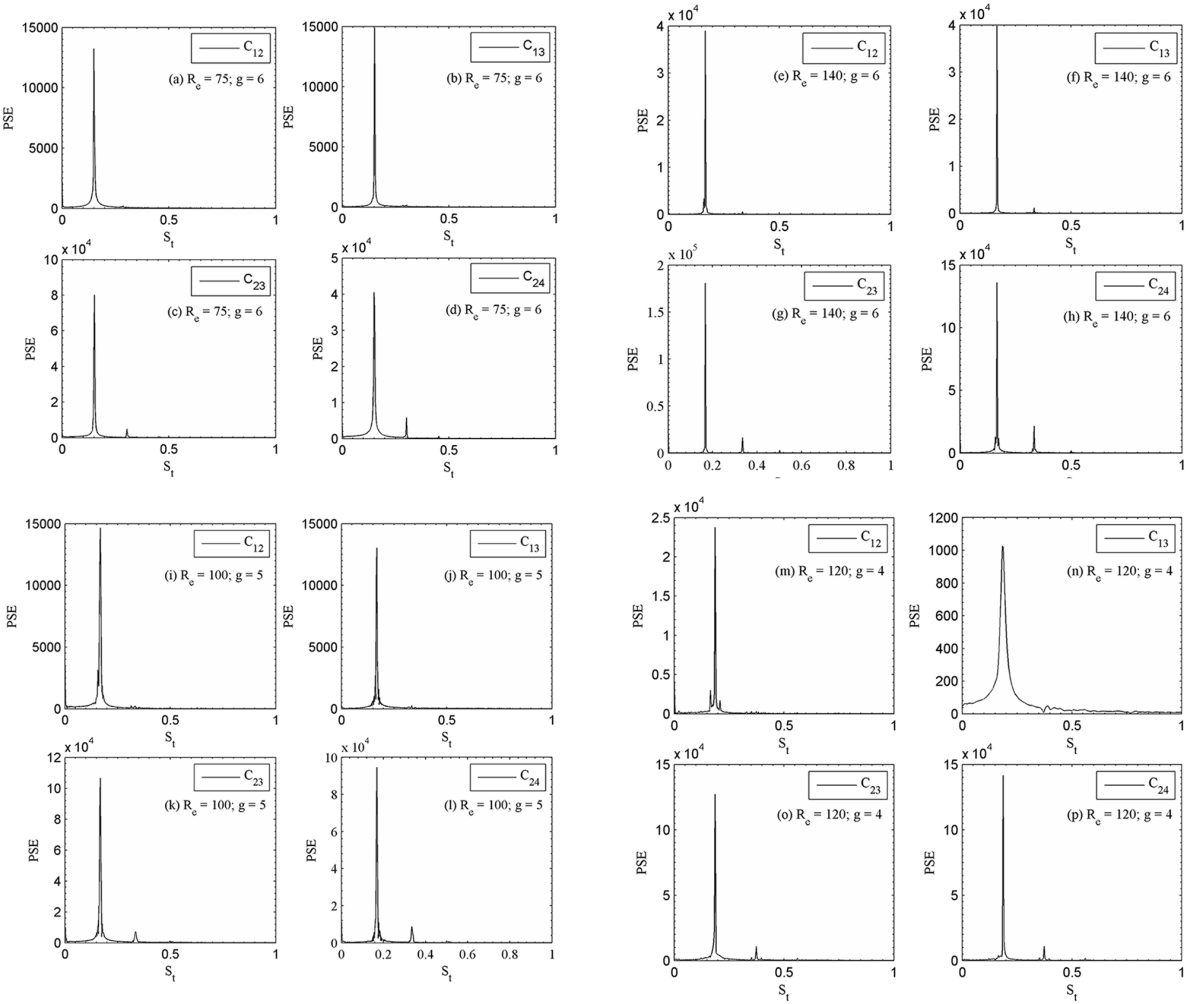
Power spectra analysis of lift coefficients (a-d) (R_e_, g) = (75, 6), (e-h) (R_e_, g) = (140, 6), (i-l) (R_e_, g) = (100, 5) and (m-p) (R_e_, g) = (120, 4).

The instantaneous vorticity contours visualization plot at (R_e_, g) = (75, 6), (140, 6), (100, 5) and (120, 4) are presented in Fig 6. For relatively large gap spacing, g = 6, 5 and 4 the shed vortices are clearly seen from both rows of the cylinders. For (R_e_, g) = (100, 5) and (120, 4), the merging of vortices observed at the downstream region; however, for (R_e_, g) = (75, 6) and (140, 6) the shed vortices throughout the computational domain almost remain distinct. Fig 6(a)–6(d) represents the change from the anti-phase to in-phase mode. At the beginning, the streets behind the cylinders are anti-phase, characterized by the same lateral spectrum between the shed vortices and same width. As a result, the vortex shedding frequencies from the cylinder differ. It is observed that anti-phase vortex shedding is predominant for larger gap spacing between the cylinders as compared to in-phase vortex shedding behind two consecutive cylinders. Williamson [35] found experimentally the anti-phase vortex shedding flow pattern for two circular cylinders at (R_e_, g) = (100, 3). Chatterjee and Biswas [16] also had similar numerical observations. The time history analysis of drag and lift coefficients are given in Fig 7(a)–7(p). By changing the Reynolds number one can clearly observe the significant variation in the signal. The lift coefficient signals in these figures clearly indicate that the secondary cylinder interaction frequency almost vanishes. The shedding pattern behind the cylinders shows some in-phase and some anti-phase characteristics. Qualitatively, the flow behavior is in good agreement with the results obtained numerically by Chatterjee and Biswas [16] for flow past two staggered rows of square cylinders. This can be further revealed from the power spectra analysis of lift coefficients presented in Fig 8(a)–8(p), where S_ts_ = 0.3047 and 0.3006 for (R_e_, g) = (75, 6) of C_23_ and C_24_, respectively (where subscript ‘s’ denotes secondary). Similarly, one can see S_ts_ for C_23_ and C_24_, respectively; in these figures. The power spectra analysis of lift coefficients clearly indicates the dominancy of primary vortex shedding frequency (S_tp_ = 0.1518 and 0.1518 for C_12_ and C_13_, respectively; for (R_e_, g) = (75, 6); where subscript ‘p’ denotes primary). This is interesting that the secondary cylinder interaction frequency disappears at the larger Reynolds number for g = 6, 5 and 4. The concept of secondary cylinder interaction frequency was proposed by Kumar *et al*. [20]. They proposed that the existence of secondary cylinder interaction frequency occurs due to narrowing and widening of the wake behind one cylinder. Chatterjee and Biswas [16] in there simulation for (R_e_, g) = (100, 5) obtained S_tp_ = 0.187, which fairly agrees with our present computed results although, the Reynolds numbers are somewhat different. The downstream row of cylinders is shedding vortices as an isolated cylinder and no sign of the frequency of oscillation of the upstream row of cylinders are noticeable in the spectra analysis of lift coefficients. Our results are in good agreement with those of the Kumar *et al*. [20] for flow past a row of square cylinders.

**Fig 9:**
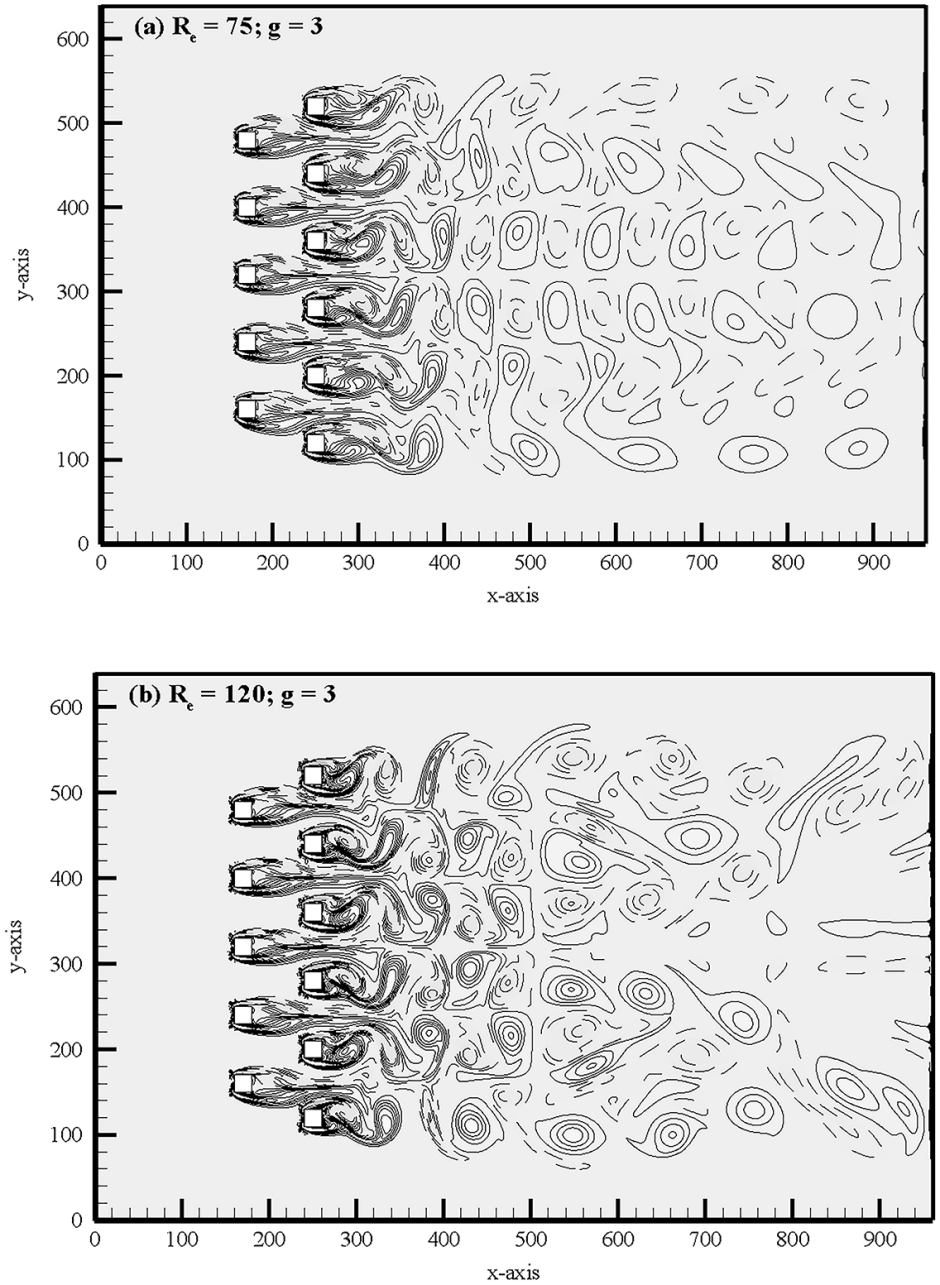
Vorticity contours visualization at (a) (R_e_, g) = (75, 3) and (b) (R_e_, g) = (120, 3).

**Fig 10:**
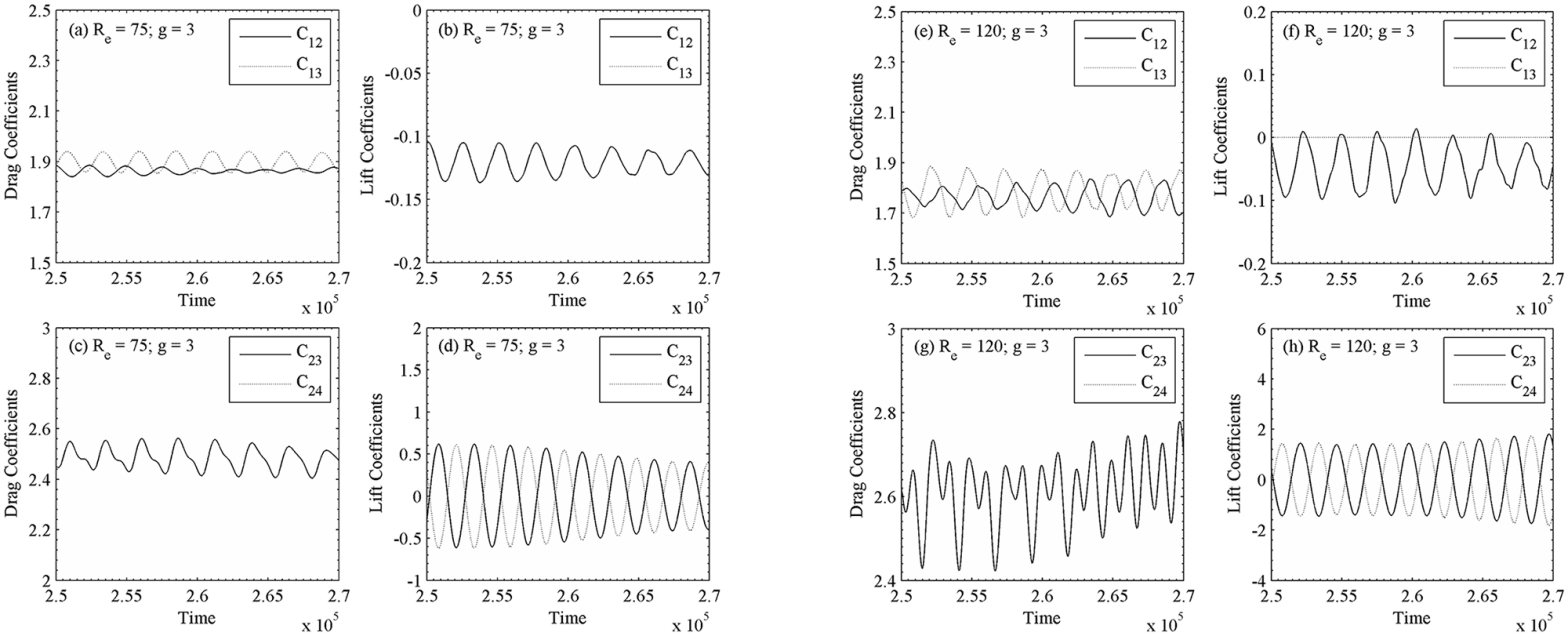
Time-trace analysis of drag and lift coefficients at (a-d) (R_e_, g) = (75, 3) and (e-h) (R_e_, g) = (120, 3).

**Fig 11:**
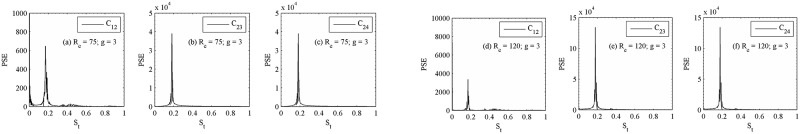
Spectra analysis of lift coefficients at (a-c) (R_e_, g) = (75, 3) and (d-f) (R_e_, g) = (120, 3).

Fig 9(a) and 9(b) shows the vorticity contours of quasi-periodic-I flow pattern. The time history analysis of lift coefficients for R_e_ = 120 at g = 3 in Fig 10(a)–10(h) also indicates the existence of secondary cylinder interaction frequency, with S_ts_ = 0.021, and primary vortex shedding frequency, with S_tp_ = 0.18 in Fig 11(a)–11(f). The time-trace analysis of lift coefficients for (R_e_, g) = (120, 3) in these figures indicates the presence of very small secondary cylinder interaction frequency. For (R_e_, g) = (75, 3) and (120, 3), we noticed that the time period of consecutive primary and secondary cycles is different and approximately constant, respectively. The very similar characteristics are observed for (R_e_, g) = (100, 3) and (140, 3). On the basis of these characteristics, we called it quasi-periodic-I flow pattern. Sewatkar *et al*. [12] had similar observations at (R_e_, g) = (100, 3) for flow past a single row of square cylinders.

**Fig 12:**
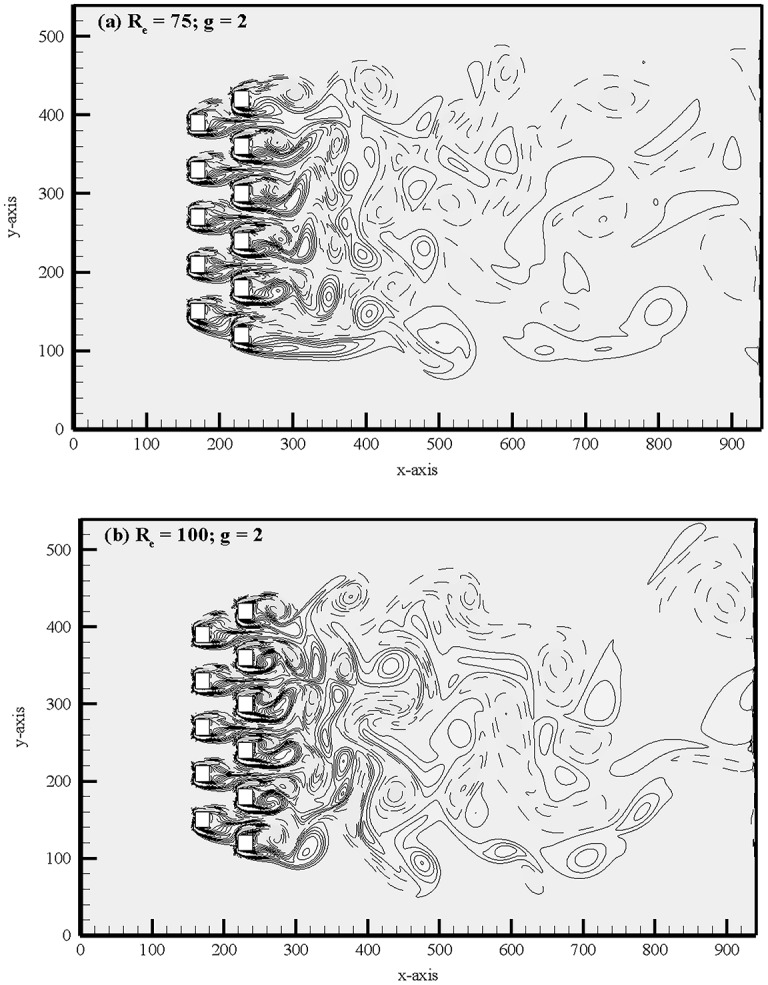
Instantaneous vorticity contours visualization at (a) (R_e_, g) = (75, 2) and (b) (R_e_, g) = (100, 2).

**Fig 13:**
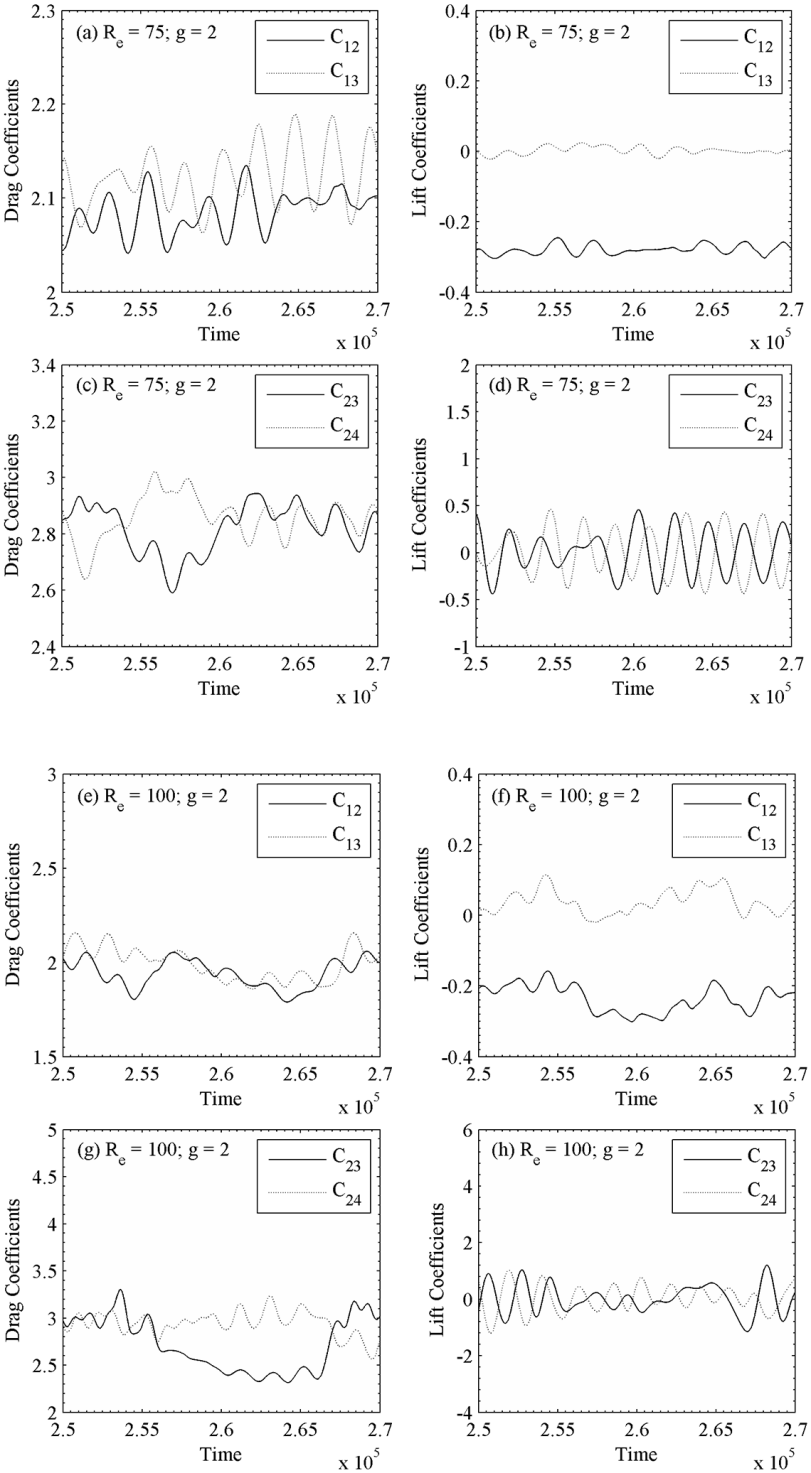
Time-history analysis of the drag and lift coefficients (a-d) (R_e_, g) = (75, 2) and (e-h) (R_e_, g) = (100, 2).

**Fig 14:**
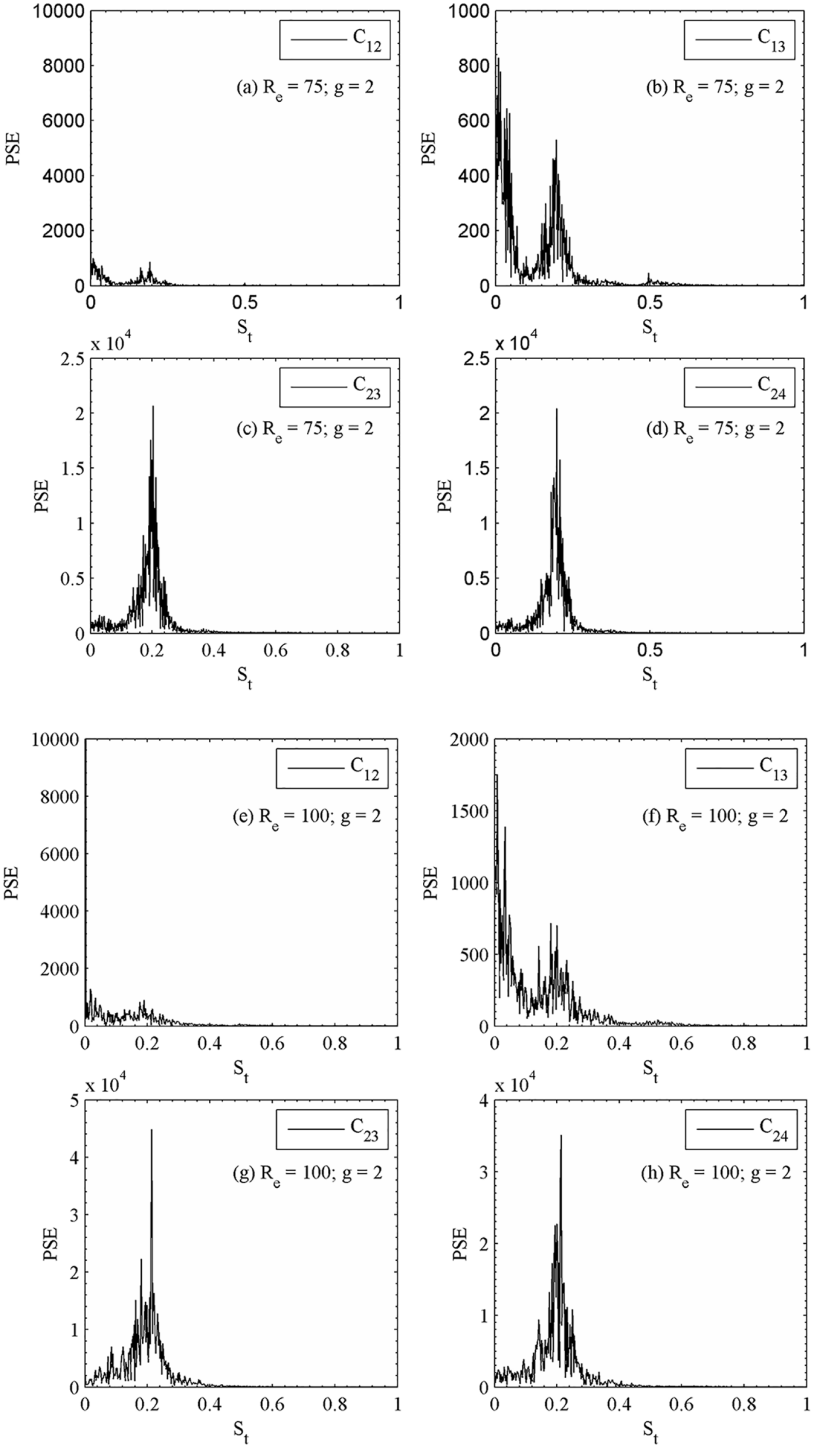
Power spectra analysis of lift coefficients (a-d) (R_e_, g) = (75, 2) and (e-h) (R_e_, g) = (100, 2).

The instantaneous vorticity contours visualization plot at g = 2 for R_e_ = 75 and 100 are shown in Fig 12(a) and 12(b). For R_e_ = 100, the shed vortices are seen approximately up to x/d ≈ 10 and then coalesces with each other shed vortices further downstream of the computational domain. One can clearly see the phase difference of shedding from an adjoining cylinder of second row. It is observed that the shedding phase difference of certain adjoining cylinders is maintained for larger time. In case of two side-by-side circular cylinders for R_e_ = 100 and 1 ≤ g ≤ 5, Williamson [35] observed that the changeover between in-phase and anti-phase vortex shedding mode lasts for a large number of vortex shedding cycles. Furthermore, at g = 2 the time history analysis of drag and lift coefficients for R_e_ = 75 in Fig 13(a)–13(d) reveals that the primary and secondary cycles are not showing constant behavior for different time periods. Similar drag and lift coefficients characteristics are observed in Fig 13(e)–13(h) for R_e_ = 100, respectively. The power spectra analysis of lift coefficients at g = 2 for different Reynolds number indicates S_tp_ = 0.0.0149, 0.0089, 0.2160 and 0.2113, and S_ts_ = 0.0208, 0.0327, 0.2101 and 0.1994 for C_12_, C_13_, C_23_ and C_24_, respectively; for R_e_ = 100. On the basis of above characteristic, the flow is so-called quasi-periodic-II flow pattern in Fig 14(a)–14(h). In quasiperiodic-II flow pattern both the primary and secondary cycles have variable time period.

**Fig 15:**
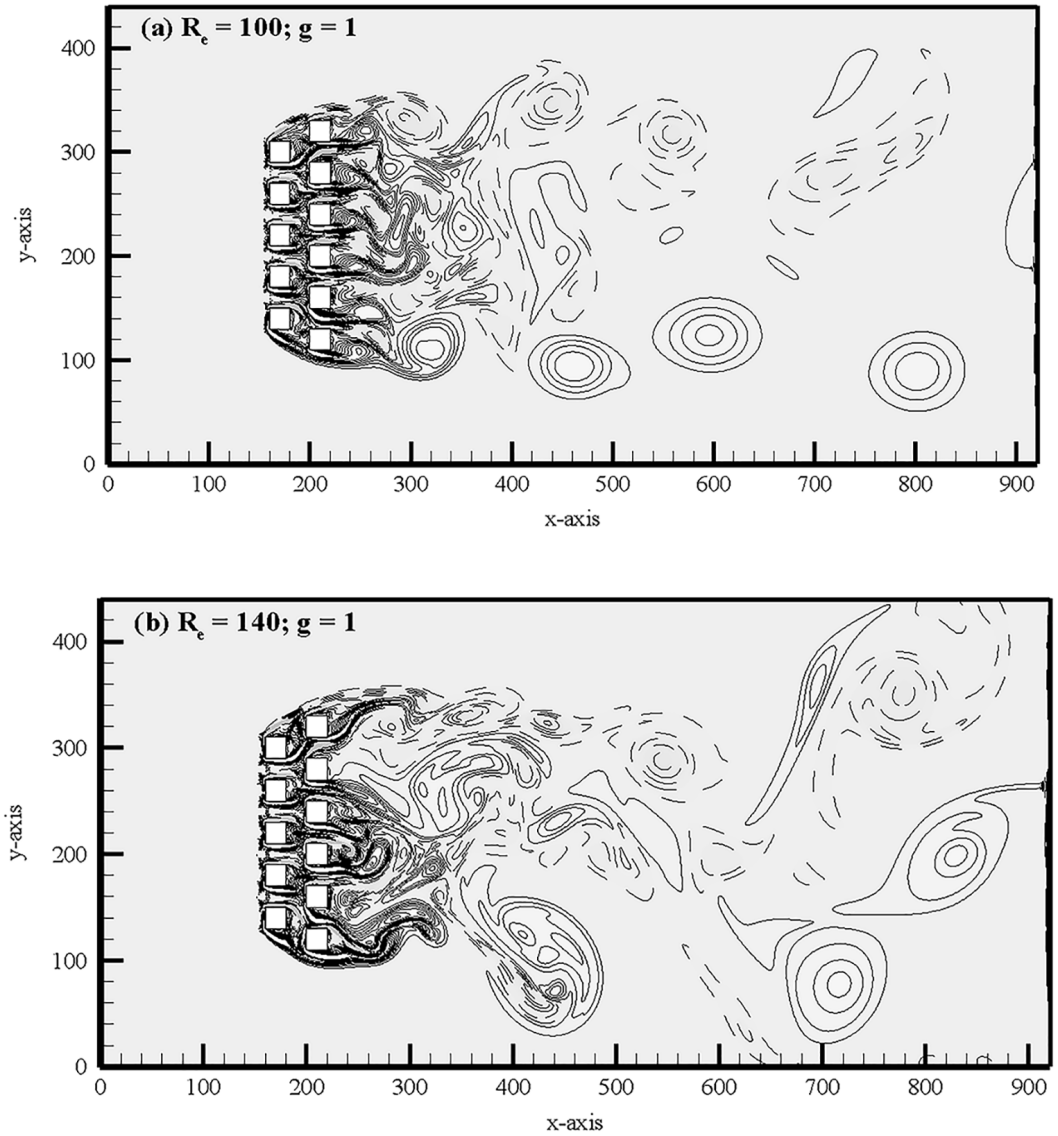
Instantaneous vorticity contours visualization at (a) (R_e_, g) = (100, 1) and (R_e_, g) = (140, 1).

**Fig 16:**
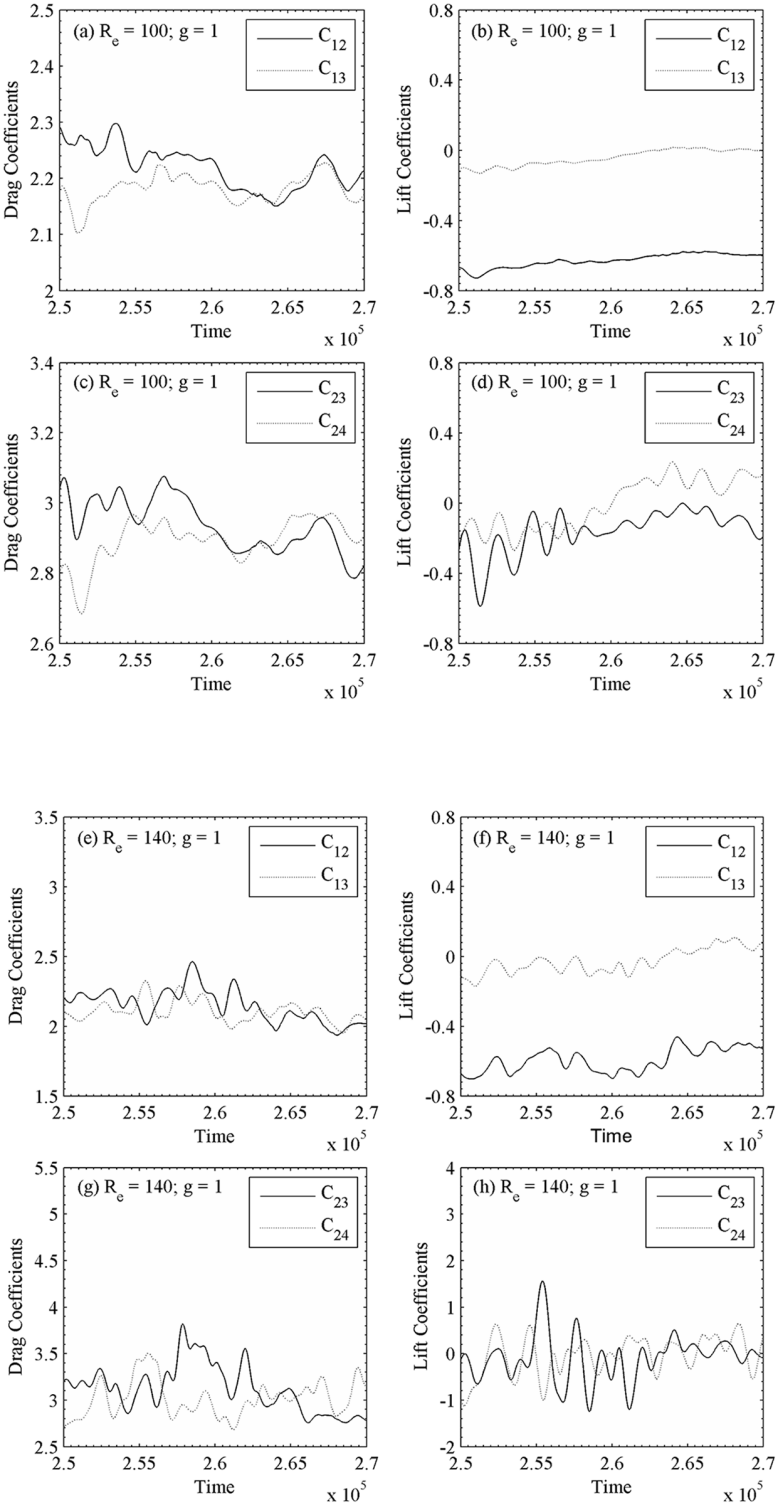
Time-history analysis of drag and lift coefficients of (a-d) (R_e_, g) = (100, 1) and (e-h) (R_e_, g) = (140, 1).

**Fig 17:**
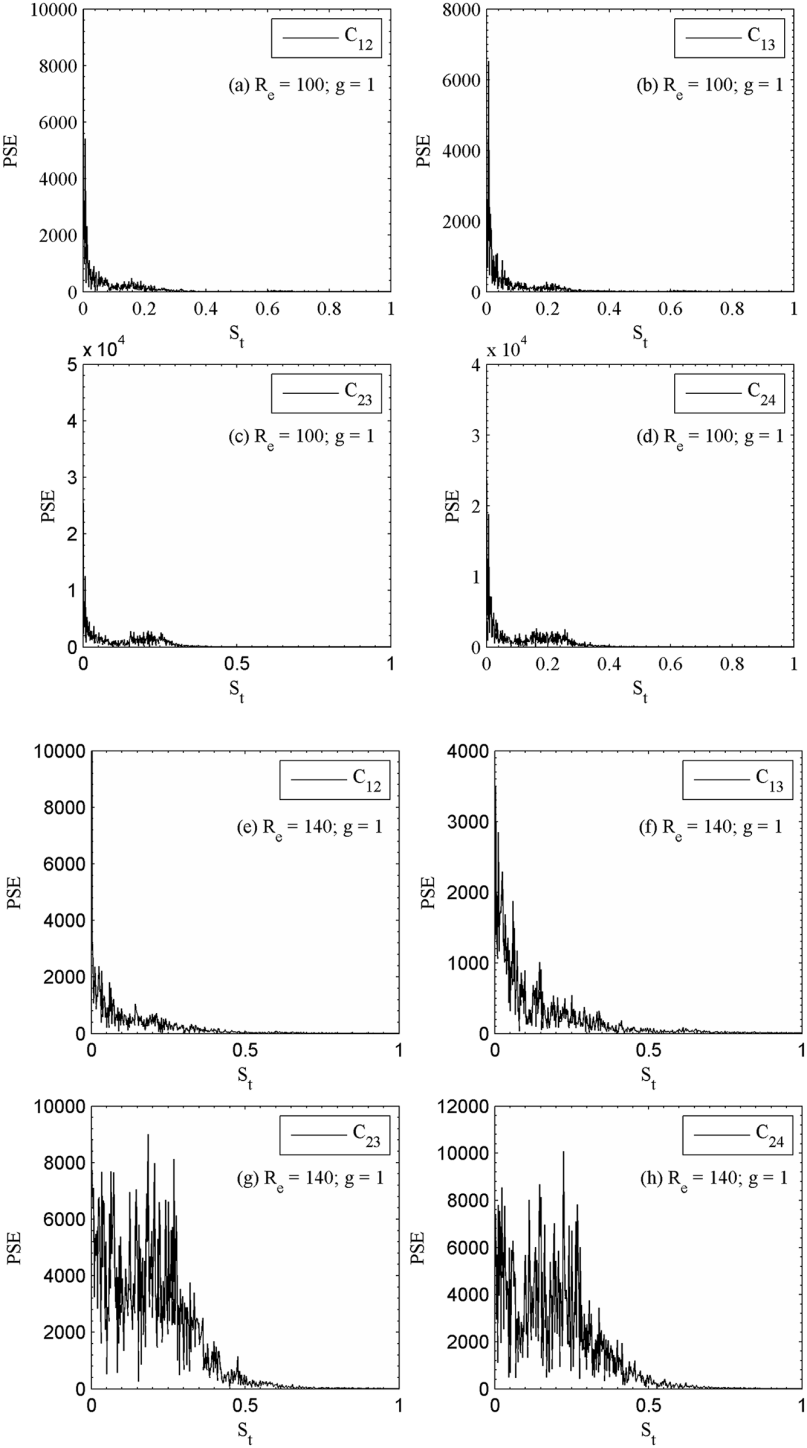
Power spectra analysis of lift coefficients (a-d) (R_e_, g) = (100, 1) and (e-h) (R_e_, g) = (140, 1).

**Fig 18:**
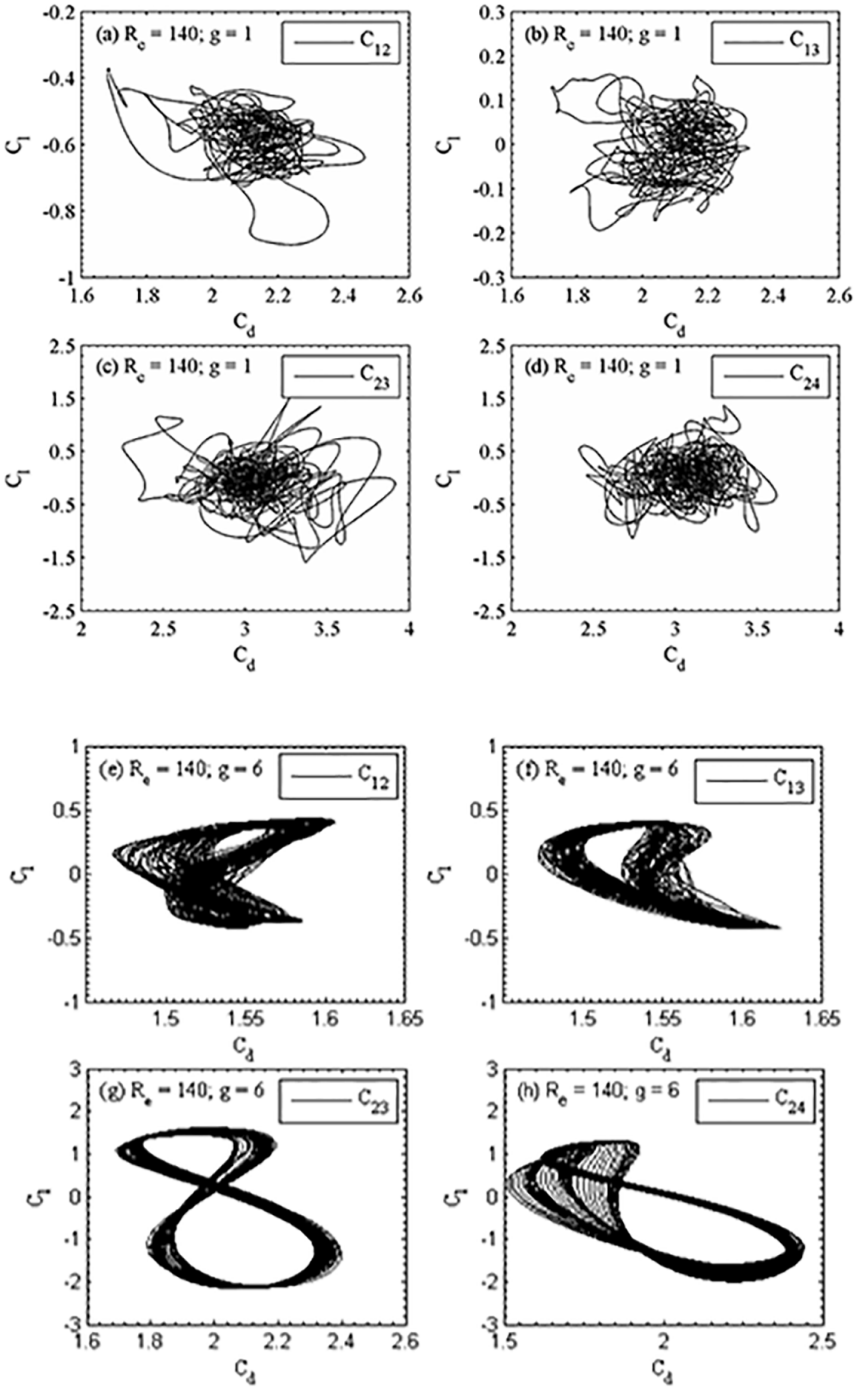
Portrait plot of the drag and lift coefficients of (a-d) (R_e_, g) = (140, 1) and (e-h) (R_e_, g) = (140, 6).

At g = 1, the instantaneous vorticity contour plots for different Reynolds numbers are shown in Fig 15(a) and 15(b). The shed vortices coalesce together just near downstream of the second row of cylinders for R_e_ = 100. This shedding coalesce occurs at small downstream of the second row of cylinders and some of the wakes appear wider and narrower. As a result, the strong merging and distortion of shed vortices clearly can be seen as they move downstream throughout the entire computational domain and the flow completely behaves chaotic. The similar characteristics observed for R_e_ = 140 in Fig 15(b). The time history analysis of drag and lift coefficients for different Reynolds numbers confirms that the flow is chaotic in nature which is presented in Fig 16(a)–16(h). The lift forces on downstream row of cylinders in these figures are very irregular and anti-phase with each other (only occasionally). It is observed for small gap spacing that the flow interference between successive cylinders is too strong. Furthermore, the jets are merging immediately behind the cylinders and also interact with the shed vortices. These observations are also verified from the time history analysis of the drag and lift coefficients, and the power spectra analysis of lift coefficients (Fig 17(a)–17(h)).The power spectra analysis of lift coefficients further reveals the behavior of chaotic flows as a result of broad and continuous spectrum. It is concluded that at lower gap spacing the shed vortices interact so strongly that the Reynolds number does not affect the basic characteristics of the flow from R_e_ = 75 to 140. We further observed for chaotic flow pattern that there is no relation between shed vortices from different cylinders and showing haphazard motion of shed vortices from the cylinders. The secondary cylinder interaction frequency completely dominates the primary vortex shedding frequency. The chaotic flow pattern was further confirmed from the phase portrait plots shown in Fig 18(a)–18(h). From these plots one can clearly see that the dynamics of the force coefficients of two adjoining cylinders progressively decouples, as suggested by Ravoux *et al*. [36]. The chaotic nature of flow is shown in the portrait diagram in these figures of drag versus lift coefficients for (R_e_, g) = (140, 1). The shed vortices behind the cylinders are strongly decoupled. However, the strong jet flow between the cylinders causes a chaotic state.

In synchronous flow pattern, the lift forces are almost symmetric, resulting in zero mean lift force as observed in portrait plots. The periodic and repulsive forces between the downstream row of cylinders have higher amplitude than those between the upstream row of cylinders. The irregularity of the lift coefficient on downstream row of cylinders is due to the jet flow between the cylinders. The trajectories in portrait plots are remains in figure-eight shape for last four figures.

**Fig 19:**
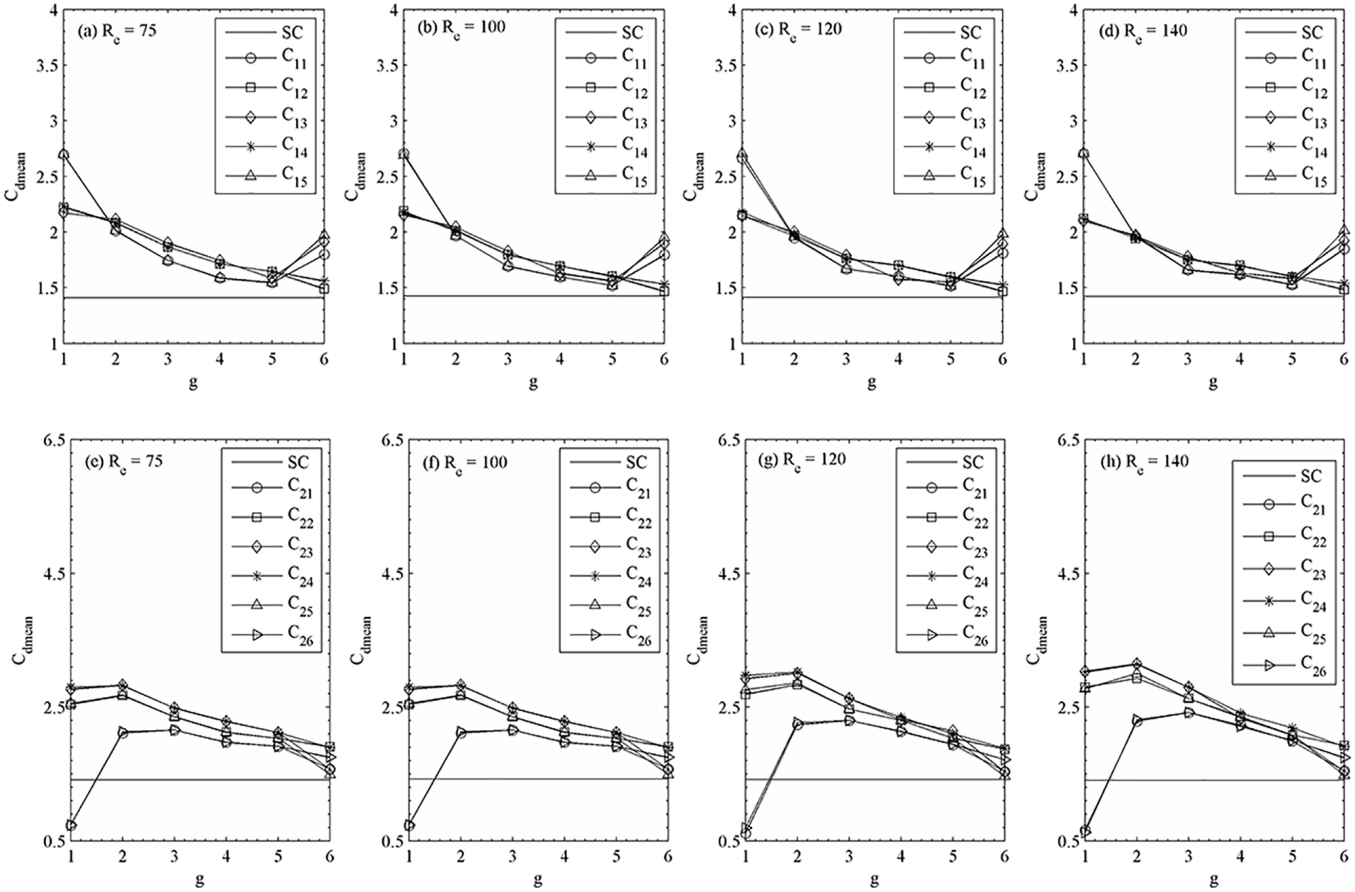
Variation in the mean drag coefficient with gap spacing at (a, c) R_e_ = 75, (b, f) R_e_ = 100, (c, g) R_e_ = 120 and (d, h) R_e_ = 140.

The relationship between the statistical characteristics and vortex patterns is another interesting issue and will be discussed in detail in this section. In coming figures the aerodynamic characteristics are presented in mean drag coefficient, Strouhal number, root-mean-square values of drag and lift forces. For comparison, the results of single isolated cylinder are also given in these figures. It is found that the C_dmean_ depends both on the R_e_ and g. The C_dmean_ of upstream and downstream row of cylinders are quite different. The C_dmean_ for the very small gap spacing (g = 1 and 2) is larger compared to the other gap spacing between the cylinders. C_dmean_ for isolated cylinder is smaller than the flow past two staggered rows of cylinders configuration, except the case of (R_e_, g) = (75–100, 1), where C_dmean_ of the cylinders (C_25_, C_26_) is lower than the value of single square cylinder. As observed in Fig 19(a)–19(h), the wake response transition behind the downstream row of cylinders leads to significant change in C_dmean_. For (R_e_, g) = (75–100, 2), a sudden jump in C_dmean_ of C_25_ and C_26_ is due to changeover of the chaotic flow pattern to quasi-periodic-II flow pattern. As the gap spacing between the cylinders is increased to g = 6 and 5, the synchronized vortex shedding behind the cylinders dominates the wakes, then the C_dmean_ of downstream row of cylinders is almost similar to that for flow past a single square cylinder. However, it is not the case for upstream row of cylinders. Compared to upstream row of cylinders the downstream row of cylinders gives quite different values.

**Fig 20:**
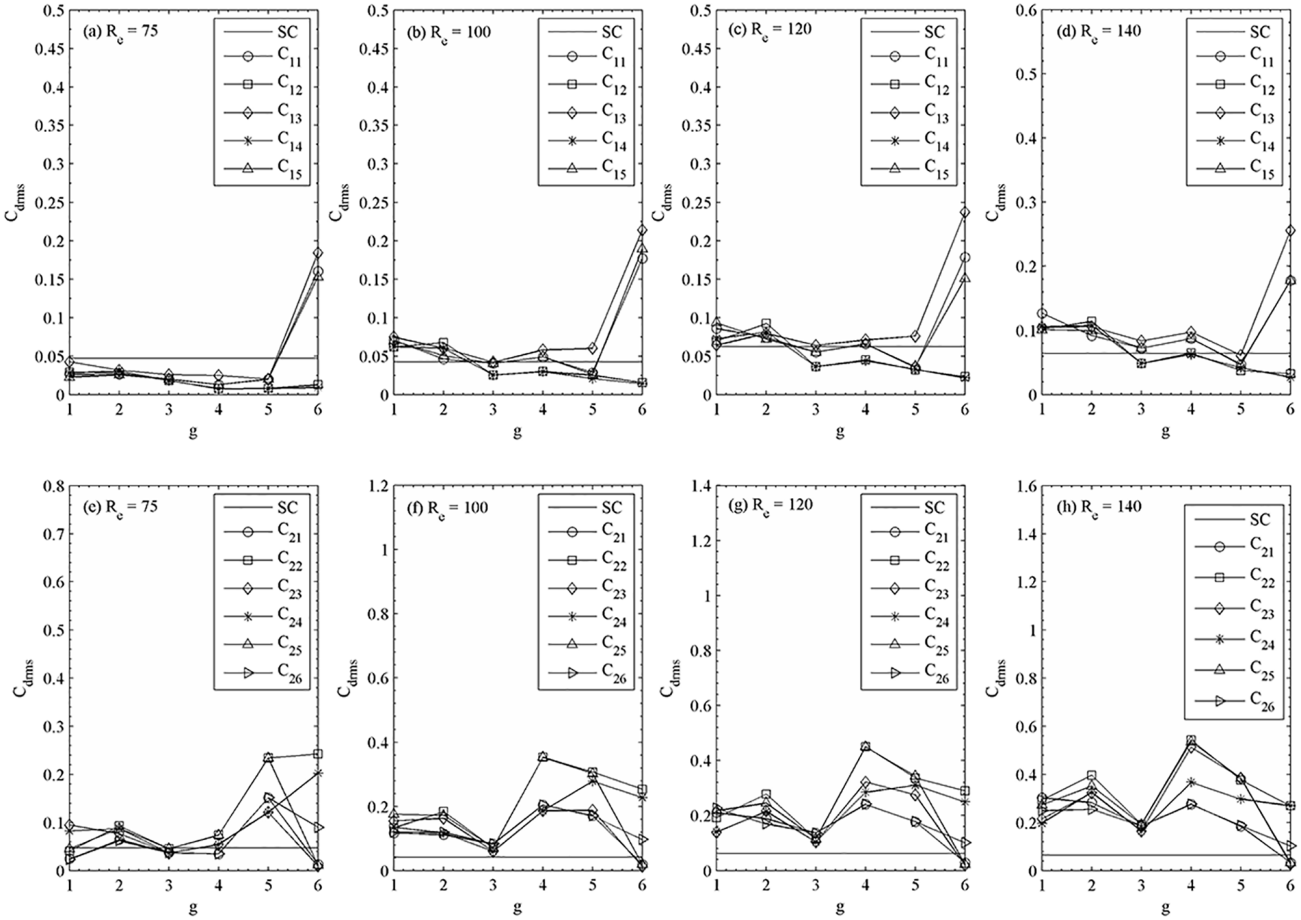
Variation in the Strouhal number with gap spacing at (a, c) R_e_ = 75, (b, f) R_e_ = 100, (c, g) R_e_ = 120 and (d, h) R_e_ = 140.

The variations of the Strouhal number with gap spacing for different Reynolds number are shown in Fig 20(a)–20(h) together with the Strouhal number of flow past a single square cylinder. In these figures, we present only the primary vortex shedding frequency values. As shown in last four figures, the vortex shedding frequency strongly depends on the gap spacing, especially at g ≤ 3. For the synchronized flow pattern, the shedding frequency is almost constant and very close to isolated cylinder value, for example, S_t_ = 0.1392, 0.1498, 0.1542, and 0.1565 at R_e_ = 75, 100, 120, and140, respectively. Sudden jumps in vortex shedding frequency are observed for quasi-periodic-II and chaotic flow patterns. We have not observed Strouhal value for those cylinders which shows steady behavior. The Strouhal number values for individual cylinder are smaller than that of a single cylinder for downstream row of cylinders at g = 1 for all chosen Reynolds numbers. On the other hand, in the case of upstream row of cylinders it is smaller at g = 2 for R_e_ = 75; g = 1 and 2 for R_e_ = 100 and 120; and g = 2 and 3 for R_e_ = 140. The discontinuities in Strouhal number occur at the Reynolds number between any two flow patterns. When the wake of the two rows of staggered square cylinders transits from chaotic flow pattern to quasi-periodic-I flow pattern at (R_e_, g) = (75, 1–2) and (100, 1–2); and from quasi-periodic-I flow pattern to quasi-periodic-II flow pattern at (R_e_, g) = (75, 2–3) and (100, 2–3), the Strouhal number shows increasing and decreasing behavior (in last four figures). The secondary cylinder interaction frequency is 0.04 for all chosen Reynolds number and is larger than the values observed at g = 6, 5, and 4 (in last four figures).

**Fig 21:**
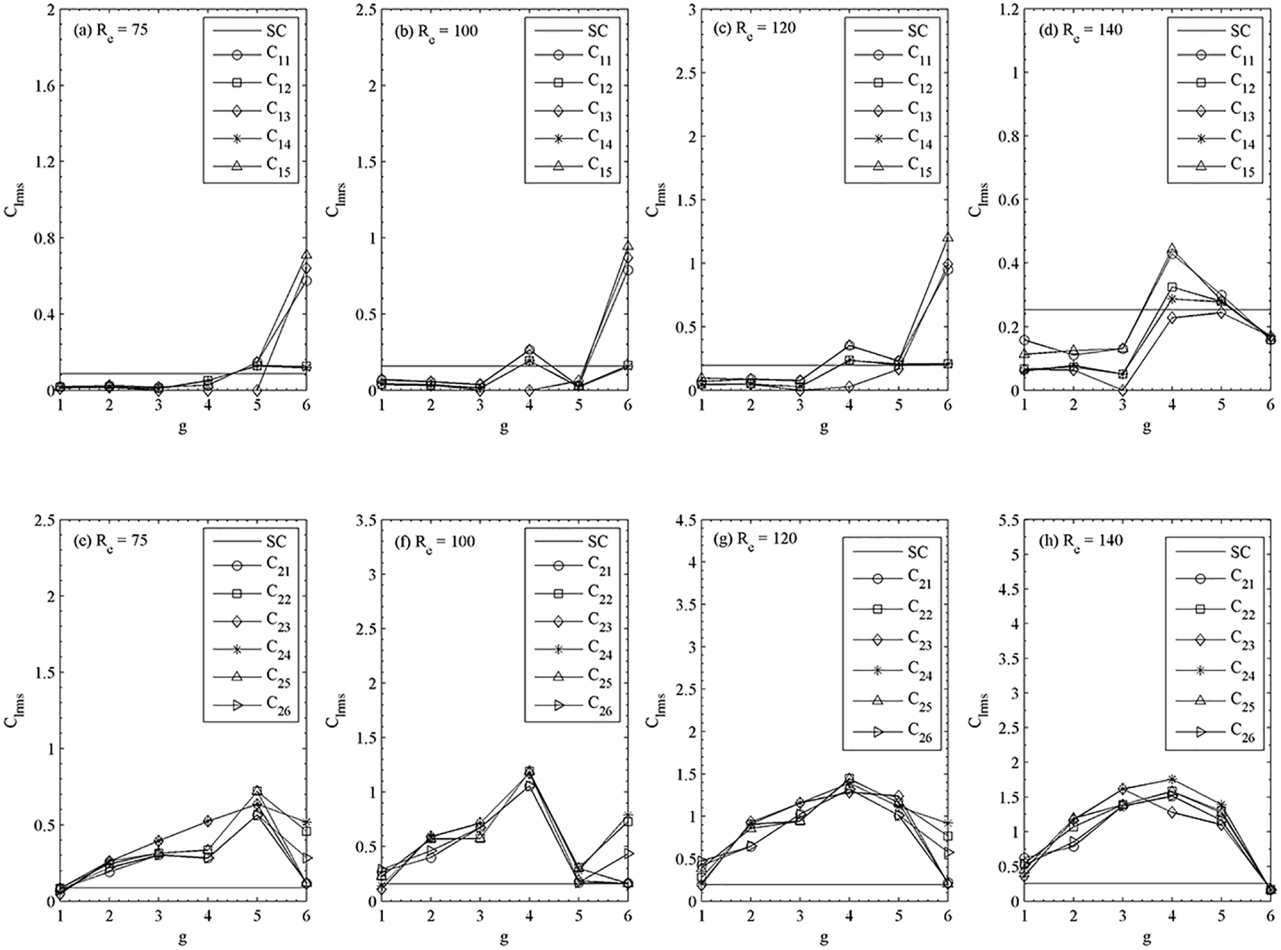
Variation in the root-mean-square value of drag coefficient with gap spacing at (a, c) R_e_ = 75, (b, f) R_e_ = 100, (c, g) R_e_ = 120 and (d, h) R_e_ = 140.

The root-mean-square values of drag coefficients are given in Fig 21(a)–21(h) together with a single square cylinder values for comparison. The increasing and decreasing behavior at relatively large gap spacing (g = 4–6) confirms that in synchronous flow patterns shed vortices behind the consecutive cylinders change their behavior from in-phase mode to anti-phase mode. Dramatic drop happens similar to that of quasi-periodic-II flow pattern at (R_e_, g) = (100, 3) and quasi-periodic-II flow pattern at (R_e_, g) = (100, 2).

**Fig 22:**
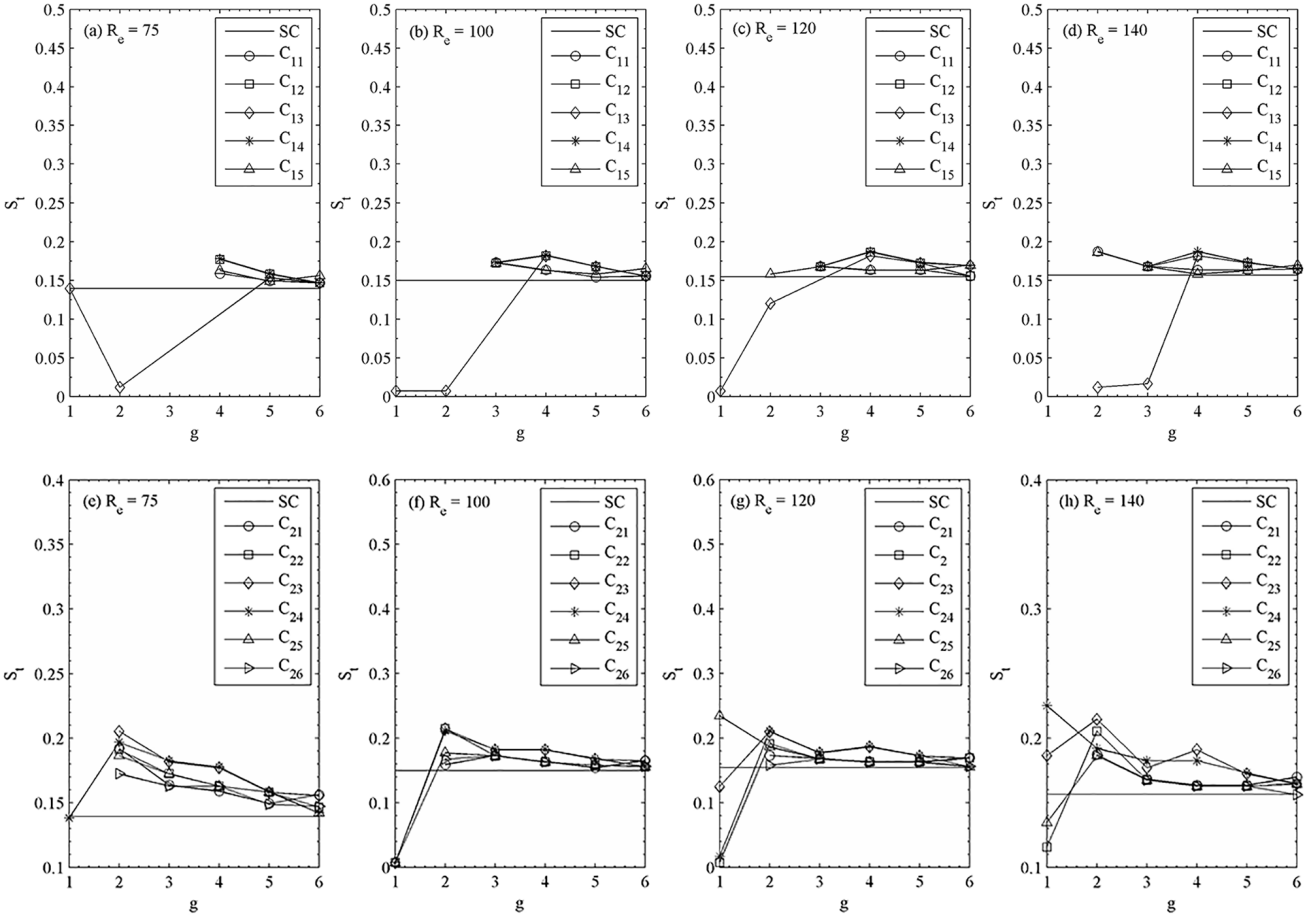
Variation in the root-mean-square value of lift coefficient with gap spacing at (a, c) R_e_ = 75, (b, f) R_e_ = 100, (c, g) R_e_ = 120 and (d, h) R_e_ = 140.

**Fig 23:**
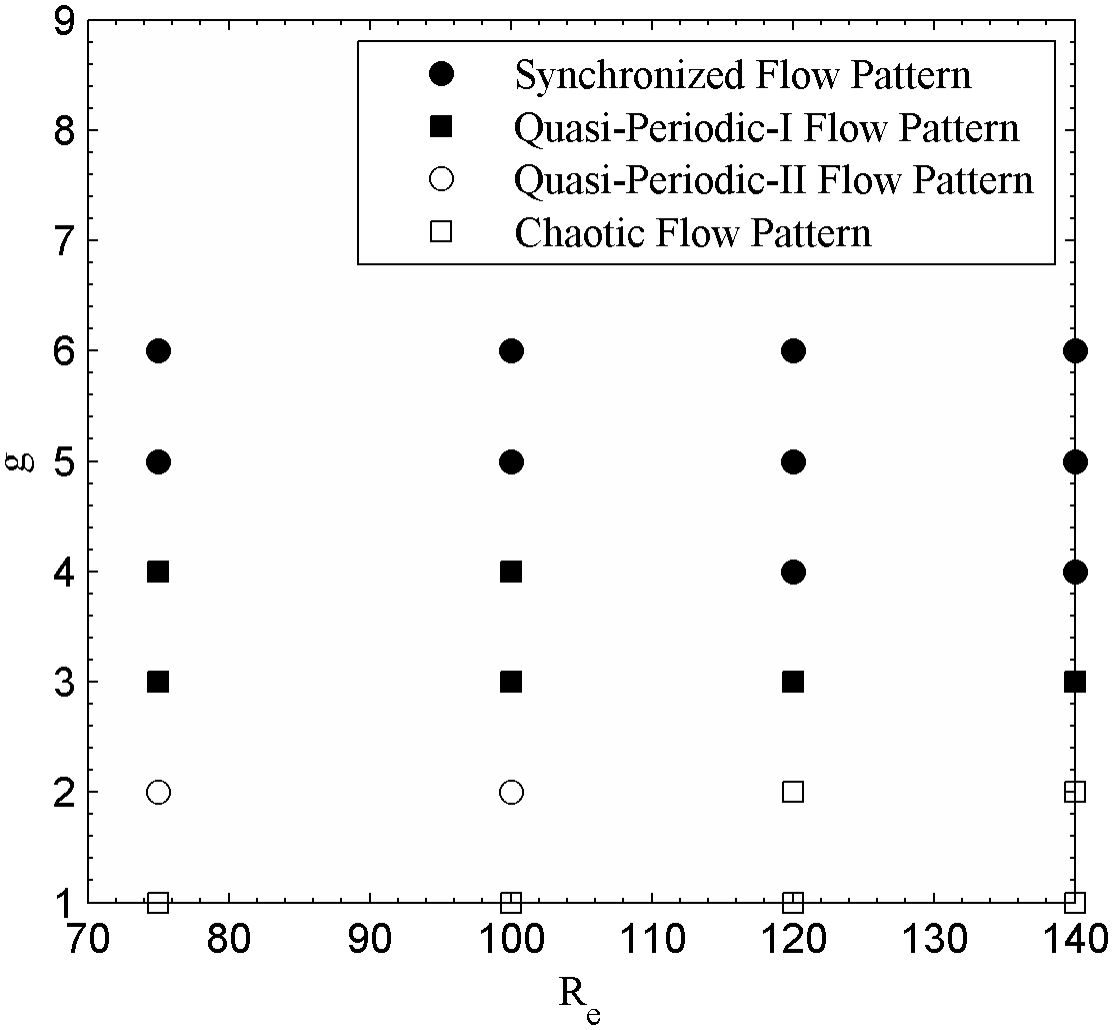
Flow patterns map at different Reynolds numbers and gap spacings.

The root-mean-square values of lift coefficients are given in Fig 22(a)–22(h). The single square cylinder values are also given in these figures for comparison. The very small C_lrms_ for g = 6, 5 and 4 indicates that the vortex shedding is very weak behind the cylinders for synchronous flow pattern. Furthermore, the C_lrms_ of downstream rows of cylinders are close to isolated cylinder with the increase of gap spacing. At all these relatively large gap spacing (g = 6, 5 and 4), we found that as the Reynolds number changes, transition in flow patterns from quasi-periodic-I to synchronous occurs (Fig 23).

The flows patterns map have been demarcated based on the characteristics observed in terms of vorticity contours visualization; time history analysis of the drag and lift coefficients; and power spectra analysis of the lift coefficients. The flow patterns map as a function of R_e_ and g is given in Fig 23. As we observed synchronous, quasiperiodic-I, quasiperiodic-II and chaotic flow patterns for 75 ≤ R_e_ ≤ 140 and 1 ≤ g ≤ 6. These flow patterns were also observed by Sewatkar *et al*. [12] and Kumar *et al*. [20] for flow past a single row of square cylinders. The synchronous flow exists at higher Reynolds number at g = 4 and larger gap spacing (g = 5, 6) for all chosen Reynolds numbers. The quasi-periodic-I flow pattern was observed at g = 4 and 3 with R_e_ ≤ 100 and R_e_ ≤ 140, respectively. Quasi-periodic-II flow pattern was found at g = 2 for Reynolds numbers 75 and 100. The figure also depicts the chaotic flow pattern for all chosen Reynolds numbers at smaller gap spacing (g = 1) and at (R_e_, g) = (120, 2) and (140, 2). The same signed vortices and its strength were found behind the second row of cylinders and approximately equal to that observed for flow past a single row of square cylinders in the synchronous and quasi-periodic-II flow patterns by Sewatkar *et al*. [12]. As a result, the shed vortices move independently behind the second row of cylinders in stream-wise direction. In Quasi-periodic-I and chaotic flow patterns significant merging of shed vortices and transverse movement were observed behind the second row of cylinders throughout the computational domain. We found that both the Reynolds number and gap spacing significantly affects the flow characteristics, with the effect of gap spacing greater than that of the Reynolds number.

## 5. Conclusions

The numerical results of two-dimensional flow across two rows of identical square cylinders in staggered arrangement are reported in this study using a lattice Boltzmann method. The main goal of the study is to systematically understand the Reynolds number effect on steady to unsteady transition and to further investigate the different flow patterns, at certain spacing. Furthermore, we study the existence of secondary cylinder interaction frequency in the time series of lifts coefficients. It is found that the critical Reynolds number, at which onset of vortex shedding occurs, increases with an increase in gap spacing between the cylinders. It is also observed that the Reynolds number have substantial effect on the flow characteristics especially at g = 4 and 2. For g = 6 and 5, the secondary cylinder interaction frequency disappears at large Reynolds numbers and the flow completely dominates by the primary vortex shedding frequency. This confirms that at such large gap spacing the wakes interaction behind the cylinders is weak and it reduces further with an increase in Reynolds number. For g = 1 and 2 the secondary cylinder interaction frequency predominates the flow and as a result strong wakes interaction are observed behind the cylinders at all Reynolds numbers.

The wake interaction mechanism is also discussed in detail. At larger gap spacing (g = 6 and 5), we found weak wakes interaction and throughout the entire computational domain between the cylinders a continuous jet forms. At relatively small gap spacing (g = 4 and 3), the wakes interaction behind the cylinders strongly depend on the Reynolds number. The wakes interaction occurs due to jets coming out of the spacing between the cylinders. In quasi-periodic-I flow pattern, the secondary cylinder interaction frequency depends on the spacing between the cylinders. In quasi-periodic-II (g = 2) flow pattern, the jets effects is stronger. In chaotic flow pattern (g = 1) the jets effect is too strong and the wakes are immediately broken behind the cylinders. The primary vortex shedding frequency is strongly affected by the secondary cylinder interaction frequency at smaller gap spacing.

Finally, in this work we clearly brought out the wakes interaction behind the cylinders and the importance of jets forms between the cylinders which were observed experimentally for higher Reynolds numbers. We also discussed in detail that the physical parameters also affected due to changeover of flow patterns. Flow around a two rows of staggered square cylinders at higher Reynolds numbers will be studied in near future using the three-dimensional lattice Boltzmann simulations.
